# Immune Checkpoint Inhibitors—Associated Cardiotoxicity

**DOI:** 10.3390/cancers14051145

**Published:** 2022-02-23

**Authors:** Chenghui Li, Sajjad A. Bhatti, Jun Ying

**Affiliations:** 1 Division of Pharmaceutical Evaluation and Policy, Department of Pharmacy Practice, College of Pharmacy, University of Arkansas for Medical Sciences, Little Rock, AR 72205, USA; 2 Internal Medicine Hematology-Oncology, College of Medicine, University of Arkansas for Medical Sciences, Little Rock, AR 72205, USA; sabhatti@uams.edu; 3 Biostatistics, College of Public Health, University of Arkansas for Medical Sciences, Little Rock, AR 72205, USA; jying@uams.edu

**Keywords:** immune checkpoint inhibitors, cardiotoxicity, cardiac adverse events, real-world database

## Abstract

**Simple Summary:**

With nonspecific activation of the immune system, immune checkpoint inhibitors (ICIs) can lead to off-target immune-related adverse events (irAEs) to every organ system. Immune-related cardiotoxicity is rare but often fatal. Large population-based studies examining different ICI-associated cardiotoxicity across cancer types and agents are limited. Using data from a large network of health care organizations, this study aims to: (1) provide an estimate of the incidence of ICI-associated cardiotoxicity, (2) to determine patient and clinical characteristics associated with the risk of developing ICI-associated cardiotoxicity, and (3) to assess the overall survival of patients experiencing ICI-associated cardiotoxicity compared to patients who did not develop cardiotoxicity after ICI use.

**Abstract:**

Large population-based studies examining differences in ICI-associated cardiotoxicity across cancer types and agents are limited. Data of 5518 cancer patients who received at least one cycle of ICIs were extracted from a large network of health care organizations. ICI treatment groups were classified by the first ICI agent(s) (ipilimumab, nivolumab, pembrolizumab, cemiplimab, avelumab, atezolizumab, or durvalumab) or its class (PD-1 inhibitors, PD-L1 inhibitors, CTLA4-inhibitors, or their combination (ipilimumab + nivolumab)). Time to first cardiac adverse event (CAE) (arrhythmia, acute myocardial infarction, myocarditis, cardiomyopathy, or pericarditis) developed within one year after ICI initiation was analyzed using a competing-risks regression model adjusting for ICI treatment groups, patient demographic and clinical characteristics, and cancer sites. By month 12, 12.5% developed cardiotoxicity. The most common cardiotoxicity was arrhythmia (9.3%) and 2.1% developed myocarditis. After adjusting for patient characteristics and cancer sites, patients who initiated on monotherapy with ipilimumab (adjusted Hazard Ratio (aHR): 2.00; 95% CI: 1.49–2.70; *p* < 0.001) or pembrolizumab (aHR: 1.21; 95% CI: 1.01–1.46; *p* = 0.040) had a higher risk of developing CAEs within one year compared to nivolumab monotherapy. Ipilimumab and pembrolizumab use may increase the risk of cardiotoxicity compared to other agents. Avelumab also estimated a highly elevated risk (aHR: 1.92; 95% CI: 0.85–4.34; *p* = 0.117) compared to nivolumab and other PD-L1 agents, although the estimate did not reach statistical significance, warranting future studies.

## 1. Introduction

Immune checkpoint inhibitors (ICIs) are monoclonal antibodies that activate T cells and initiate an adaptive immune response, thereby allowing the immune system to recognize abnormal cancerous cells [1]. Currently, there are seven FDA-approved ICIs targeting three pathways: cytotoxic T-lymphocyte associated-antigen-4 (CTLA-4) (ipilimumab), programmed death receptor-1 (PD-1) (pembrolizumab, nivolumab, and cemiplimab), and programmed death-ligand-1 (PD-L1) (atezolizumab, avelumab, and durvalumab) [2]. Durable tumor responses and improvement in overall survival have been shown in numerous randomized clinical trials in patients treated with ICIs [1]. It was estimated that 43.6% of cancer patients in the US were eligible for ICI therapy in 2018 [3]. Since then, the FDA has approved more indications for ICIs [2] and the number of eligible patients is likely to be even higher. However, not all patients respond to ICIs and the overall response rate (ORR) varies by tumor type and agent [3], ranging from 10.9% for single-agent ipilimumab in previous treated melanoma [4] to 69% for pembrolizumab in relapsed/refractory classic Hodgkin’s lymphoma [5], with a subset of patients developing resistance over time [1]. To increase response and combat resistance, combinations of ICIs with each other or with treatments such as chemotherapy, radiation, and targeted therapy are increasingly being used which increases the complexity of toxicity [6]. The use of ICIs is rapidly expanding. According to the 2018 estimates by Allied Market Research, globally, the ICI market was valued at $29.8 billion in 2020, and is projected to reach $140.9 billion by 2030, at a compounded annual growth rate of 16.8% between these years [7].

With nonspecific activation of the immune system, ICIs can lead to off-target immune-related adverse events (irAEs) in every organ system [8]. It is estimated that up to 90% of patients experienced any clinically detectable irAEs and as many as 45% of patients experienced severe irAEs (grades 3–4), although the estimates vary by agents and are higher among combination therapy [9]. Immune-related cardiotoxicity is rare but often fatal [10]. The most commonly reported cardiac irAE from ICIs is myocarditis, an inflammatory disease of the heart muscle cells [10]. Existing studies have reported a wide range of incidence of ICI-associated myocarditis, ranging from 0.06% for single-agent nivolumab and 0.27% for a nivolumab and ipilimumab combination in the Bristol Myers Squibb corporate safety database [11] to 1.14% across multiple ICIs and combinations in a single-center study [12]. Both studies were conducted when ICI-associated cardiotoxicity began to be recognized [11,12]. Pharmacovigilance studies have since raised its awareness [13,14], leading to more reported cases in recent years [15]. However, diagnosis of myocarditis is challenging due to its heterogeneous clinical manifestations, which range from no symptoms with an abnormal biomarker, to nonspecific symptoms such as fatigue, to fulminant presentation with hemodynamic compromise [1,16]. Moreover, to confirm diagnosis, the gold standard is endomyocardial biopsy, which is an invasive procedure and not often performed in clinical practice [16]. Thus, the incidence of ICI-associated myocarditis is likely to be underestimated. Unlike general myocarditis, ICI-associated myocarditis is highly fatal, with a reported mortality rate of 40–50% [13,17]. In addition to myocarditis, studies of safety databases have reported other potential ICI-associated cardiotoxicity, including cardiomyopathy, conduction defects (heart block), atrial and ventricular arrhythmias, and pericarditis/pericardial effusions [18]. However, incidences of these other ICI-associated cardiotoxicities are rarely reported and limited to mostly case reports or case series [14]. Large population-based epidemiology studies are limited [19].

Using data from a large network of health care organizations, this study aims to: (1) provide an estimate of the incidence of ICI-associated cardiotoxicity, (2) to determine patient and clinical characteristics associated with the risk of developing ICI-associated cardiotoxicity, and (3) to assess the overall survival of patients experiencing ICI-associated cardiotoxicity compared to patients who did not develop cardiotoxicity after ICI use. Besides the large sample size (5518 patients), this study used newer data that cover multiple cancer sites and all seven FDA-approved agents.

## 2. Materials and Methods

### 2.1. Data Source

Data were from health care organizations (HCOs) in the research network of TriNetX. TriNetX (www.trinetx.com; accessed on 17 February 2022) is a global federated health research network that provides real-time access to data from electronic medical records (EMRs), including demographics, diagnoses, procedures, medications, laboratory testing, vital signs, and genomic information. The network consists of academic medical centers, community hospitals, and physician practices. A PubMed search on 10 January 2022 found 119 publications using TriNetX databases. More details on how TriNetX standardizes data from contributing HCOs can be found in Harrison et al. (2020) [20]. Our institution is a contributing member of the network. We queried the database on 28 October 2019 via TriNetX’s browser-based interface of all patients with a record for ICIs. At the time of the query, there were 22 HCOs in the network with relevant data. We received a de-identified patient-level dataset for this analysis. Since the dataset is de-identified, our institution’s Internal Review Board (IRB) determined that this study is not a human-subject study. (IRB #263203).

### 2.2. Patient Selection

**Figure 1:**
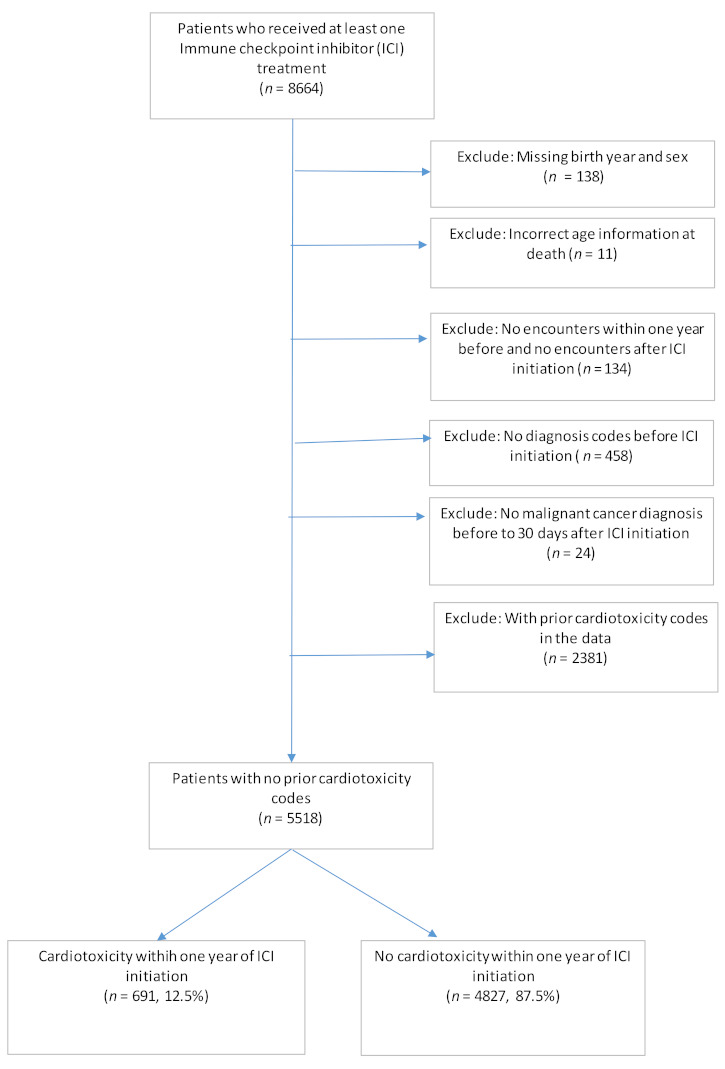
Patient selection flowchart.

Patients who received at least one treatment with an ICI (CTLA-4 inhibitors: ipilimumab (Yervoy); PD-1 inhibitors: nivolumab (Opdivo), pembrolizumab (Keytruda), and cemiplimab (Libtayo); and PD-L1 inhibitors: avelumab (Bavencio), atezolizumab (Tecentriq), and durvalumab (Imfinzi)) by 28 October 2019 were included in the study. Users of ICIs were identified using medication and procedure files. The specific ICI agents administered during visits to HCOs were coded in the Procedure file using the Healthcare Common Procedure Coding System (HCPCS) codes. Produced by the Centers for Medicare and Medicaid Services (CMS), HCPCS is a collection of standardized codes that represent medical procedures, supplies, products, and services [21]. The medication file includes medications ordered, prescribed or administered to a patient, including mediations reported by patients in the medication list of the EMR. We searched for the specific ICI agents by name in the medication file. To ensure these medications were administered (rather than just ordered or prescribed), we required either a procedure code for specific ICI agents, or a general procedure code for chemotherapy administration in the procedure file on the same day. Therefore, we ascertained the ICI administration by (1) ICI-related HCPCS codes in the procedure file and/or (2) ICI agents identified from the medication file accompanied by a CPT/HCPCS code for chemotherapy administration or HCPCS code J9999 (antineoplastic drugs, not otherwise classified) in the procedure file on the same day. (See Supplemental Table S1 for the list of procedure codes used). This resulted in 8664 patients. The date of the first ICI administration was defined as the index date. From this cohort, we further applied the following exclusion criteria: (1) missing information on birth year or gender; (2) inconsistent information on age at death; (3) no encounters within 1 year before the index date and no encounters after the index date; (4) no diagnosis codes before or on the index date; (5) no documented non-benign cancer diagnoses before the index date to 30 days after the index date; (6) had relevant cardiac adverse event (CAE) codes prior to or on the index date (see next section on CAE for details). The final sample included 5518 patients (see Figure 1 for patient selection flowchart).

### 2.3. ICI Treatment Group

ICI treatment groups were defined based on the first ICI agent(s) used by each patient. We studied these treatment groups by specific agents (nivolumab (reference group), ipilimumab, pembrolizumab, cemiplimab, avelumab, atezolizumab, durvalumab, or combination therapy), as well as by class (PD-1 inhibitors (reference group), PD-L1 inhibitors, CTLA4-inhibitors, or combination therapy). Nearly all combination therapies were for nivolumab plus ipilimumab. One patient used pembrolizumab plus ipilimumab and one used pembrolizumab plus nivolumab. These combinations were not FDA-approved therapies and may have been used in unique clinical scenarios. Therefore, we excluded these two patients from the analyses.

### 2.4. ICI-Associated Cardiotoxicity

The primary outcome was the ICI-associated cardiotoxicity defined as CAEs diagnosed within one year after ICI initiation. Patients were followed from the index date onward until 12 months, death, or the patient’s last encounter date in the database, whichever occurred earlier, to observe any CAEs. Potential CAEs were defined as new diagnoses of arrhythmia, acute myocardial infarction (AMI), myocarditis, cardiomyopathy, or pericarditis. The list of ICD-9 and 10 codes used to identify these potential CAEs were from Cathcart-Rake et al., 2020 [22] According to the authors, these conditions and corresponding codes were derived after a thorough review of irAEs noted in immunotherapy clinical trials, chemotherapy clinical trials, and case reports [22]. To reduce the risk of misclassification due to preexisting conditions, only new diagnoses of these conditions were considered and patients who had a relevant diagnosis code from the list before ICI initiation were excluded. Time to first CAE within one year after ICI initiation was calculated for patients who experienced these events. For patients who did not develop CAEs within one year after ICI initiation, their time to the first CAE was censored at 12 months, death, or their date of last contact in the database, whichever occurred first.

### 2.5. Overall Survival

The secondary outcome was death from any causes during the study period. To protect patients’ privacy, the de-identified data set we received included only patients’ ages at death without the exact dates of death. A patient was considered to have died if the age at death was reported. If a patient died, the last encounter date was used to impute for the date of death. Time from ICI initiation to death was analyzed. For patients who did not die during our study period, their time to death was censored at the date of last contact in the database.

### 2.6. Other irAEs

In addition to cardiotoxicity, ICIs may cause irAEs in other organ systems. We determined the occurrence of irAEs in other major organ systems (hematologic (anemia, thrombocytopenia, leukopenia), pneumonitis, endocrine (hypothyroidism, hyperthyroidism, hypophysitis/PGA, Hyper/hypoparathyroidism, diabetes type I, dysfunctional uterine bleeding/infertility), renal (acute kidney injury/AKI), neurological (encephalitis/myelitis/encephalomyelitis, neuritis, meningitis), hepatic (hepatitis), gastrointestinal (GI) (colitis, pancreatitis, mucositis), and skin (vitiligo)) within one year of ICI initiation. The list of ICD-9 and 10 codes used to identify these potential CAEs were from Cathcart-Rake et al., 2020, who developed these codes from a thorough review of irAEs noted in immunotherapy clinical trials, chemotherapy clinical trials, and case reports [22].

### 2.7. Patient Demographic and Clinical Characteristics

Patient demographic and clinical characteristics included age on the index date, gender, ethnicity, race, comorbidities, primary cancer site, type of first ICI treatment, and any prior cancer treatment (chemotherapy, radiation). Cancer sites included all sites with an FDA-approved ICI-indication (melanoma, lung, renal cell carcinoma (RCC), urothelial (urethra/bladder/ureter), Hodgkin’s lymphoma, Merkel cell carcinoma (MCC), gastric, colon, breast, cervical, primary mediastinal (thymic) large B-cell lymphoma), and other (include all other cancer sites) based on diagnoses codes before ICI initiation to 30 days after. Comorbidity burden was measured using the Deyo–Charlson comorbidity index based on diagnoses reported prior to or on the index date. We used the enhanced ICD-9 codes and ICD-10 codes developed and validated for consistency by Quan, 2005 [23]. The enhanced ICD-9 codes were more consistent with the ICI-10 codes and performed slightly better than the original ICD-9 codes in predicting in-hospital mortality [23]. Diagnoses of neoplasms were not included in the calculation of the index since all our subjects had cancer. However, metastatic/secondary cancers were included. Hierarchy coding was applied to prevent duplicated accounting: if a person had diagnoses of both a mild and a severe form of the disease prior to ICI use (e.g., mild and moderate/severe liver disease, diabetes with and without chronic complications), the patient was only scored on the more severe disease in the CCI [24]. Chemotherapy included all systemic agents (oral chemotherapy agents were not considered) identified using HCPCS codes in the procedure file. Radiation therapy included any radiation therapy identified using procedure codes for radiation treatment delivery (CPT/HCPCS codes) in the procedure file. Stage information was only available for a small subset of patients and, therefore, was not included in the analysis. However, during the study period, ICIs were approved to treat advanced cancers and, in our study cohort, 86% had metastatic diagnoses prior to or on the index date.

### 2.8. Statistical Analysis

Patients’ demographic and clinical characteristics were summarized using frequencies and means (standard deviations). These characteristics were compared between patients who developed CAEs within one year post ICI initiation and those who did not using t-tests for continuous variables and Chi-square tests for categorical variables. These characteristics by first ICI agent(s) and by primary cancer sites were summarized using frequencies (Supplemental Tables S2 and S3). We calculated the proportion of patients who developed any CAEs as well as proportions of patients who developed each CAE during the 12 months after ICI initiation. To account for differences in follow-up periods, we also reported the rates of CAEs per person/year and their 95% confidence intervals (95% CI), calculated using the quadratic approximation to the Poisson log likelihood for the log-rate parameter [25].

Time to first CAE within one year after ICI initiation was analyzed using survival analysis. Although Kaplan–Meier (KM) method and Cox proportional hazard (PH) models are often used to estimate survival and associated hazard ratios (HRs), these methods treat death as an uninformative censoring event [26]. In our study population of patients with advanced stage cancers, the mortality rate was high (29.1% died within one year of ICI initiation). This creates competing risk for CAEs (i.e., a patient died before experiencing CAEs). In the presence of competing risk, the KM method overestimates the cumulative incidence rate [26]. Therefore, we estimated cumulative incident rates and adjusted hazard ratios (aHRs) of CAEs using the competing-risks regression model according to the method of Fine and Gray (1999) [27]. Use of the Fine–Gray sub-distribution hazard model is recommended when the focus is on estimating incidence or predicting prognosis in the presence of competing risks [26]. “Failure to account correctly for competing events can result in adverse consequences, including overestimation of the probability of the occurrence of the event and misestimation of the magnitude of relative effects of covariates on the incidence of the outcome” [26]. Two regression analyses were conducted to assess the association with: (1) different ICI agent(s); and (2) different ICI classes (PD-1 inhibitors, PD-L1 inhibitors, CTLA4-inhibitors, or their combination (ipilimumab + nivolumab)). Both models adjusted for differences in patient demographics (age, gender, race, ethnicity), comorbidity index, pre-existing cardiovascular conditions (hypertension, cerebrovascular disease (CED), congestive heart failure (CHF), myocardial infarction (MI), peripheral vascular disease (PVD)), renal disease, moderate/severe liver disease, and major (≥30 cases in CAE and non-CAE groups) cancer sites with FDA indication for ICI use by 2018 (lung, melanoma, RCC, urothelial, head and neck, MCC, and liver). Although MCC is a rare cancer, its mortality is high [28]. We included MCC as a covariate so that it would not bias the estimates of the major cancer sites when compared to other cancers. Liver cancer was also included because the proportion of patients with liver cancer was statistically significant between the CAE and non-CAE groups in the bivariate analysis. After each regression, we presented the adjusted cumulative incident curves by first ICI agent(s) and ICI class, respectively.

For overall survival, we used KM survival curves to summarize time to death due to all causes after ICI initiation. The aHRs from a multivariate Cox’s PH regression model were used to compare overall survivals between patients who developed CAEs within one year against those who did not. For this analysis, a binary variable indicating whether a patient developed CAEs within one year after ICI initiation (Y/N) was included as a covariate in addition to the aforementioned covariates. Considering the Schoenfeld residuals-based test [29], which showed a significant violation of the PH assumption (*p* = 0.0033), we decided to use a time-varying Cox regression model by adding an interaction term of time with the indicator variable, the coefficient of which revealed how the HR changed over time between patients who developed CAEs within one year and those who did not. As previously described, myocarditis is a rare but highly fatal CAE. For patients who developed myocarditis within one year and died afterwards (*n* = 55), we also graphically presented the time to first myocarditis diagnosis and time to death after myocarditis diagnosis for each patient to graphically show the distributions of each variable and examine if they were correlated (i.e., whether time to death after myocarditis depended on how soon a patient developed myocarditis after ICI initiation). 

We used SAS version 9.4 (SAS Institute Inc., Cary, NC, USA) and Stata version 17 (Stata Corp, College Station, TX, USA) for all analyses.

## 3. Results

After applying the inclusion and exclusion criteria, the final data set included 5518 patients who received at least one cycle of ICI treatment and had no prior documented cardiotoxicity codes. Among them, 691 developed CAEs within one year of ICI initiation (12.5%) (Figure 1).

Table 1 reports patients’ demographic and clinical characteristics. Compared to patients who did not develop CAEs within one year of ICI initiation, patients who developed CAEs were older (Age ≥ 65 years 54.6% vs. 44%, *p* < 0.0001), more likely to be males (63.2% vs. 56.7%, *p* = 0.0012), less likely to be Hispanic (1.4% vs. 3.4%, *p* = 0.0144), had higher comorbidity burdens (*p* = 0.0042), and were more likely to have pre-existing cardiovascular diseases (CED: 12.9 vs. 10.3%, *p* = 0.0375; CHF: 5.9% vs. 2.6%, *p* < 0.001; MI: 4.8% vs. 2.3%, *p* = 0.0002; PVD, 15.6% vs. 12.3%, *p* = 0.0129; and hypertension, 50.8% vs. 45.1%, *p* = 0.0046) or renal disease (RD: 13.7% vs. 10.8%, *p* = 0.0221)). Lung (35.7%) and melanoma (33.7%) were the most common cancer sites. Patients who developed CAEs within one year of ICI initiation were more likely to have lung cancer (42.3% vs. 34.7%, *p* < 0.0001) and less likely to have liver cancer (2.8% vs. 6.6%, *p* < 0.0001) compared to those who did not. About one third of patients received some radiation treatment and 22% had received chemotherapy prior to ICI initiation. There were no differences in these prior treatments between patients who developed CAEs within one year of ICI initiation and those who did not (*p* > 0.05). The most common chemotherapy agents used were alkylating agents (84% of patients who received any chemotherapy prior to ICI initiation). Presence of irAEs in other organ systems during the 12 months after ICI initiation was high, ranging from 7.6% for skin to 44.9% for endocrine. Patients who developed CAEs within one year were also more likely to experience those other irAEs compared to patients who did not (all *p* < 0.05), except for skin irAEs (*p* = 0.3160).

Table 2 reports the ICI treatment received by patients. Patients who developed CAEs within one year of ICI initiation were more likely to have received ipilimumab monotherapy (12.3% vs. 8.0%) as their first ICI treatment whereas those who did not develop CAEs were more likely to have initiated on nivolumab monotherapy (28.4% vs. 32.5%) or its combination with ipilimumab (6.9% vs. 8.7%). The overall difference in the first ICI treatments between the two groups was statistically significant (*p* = 0.0038). During the study period, the majority (84%) received treatment with only one ICI agent but 16% received two or three different ICI agents (switched or used in combination therapies) during the study period. There was no difference in the number of ICI agents used between patients who developed CAEs within one year after ICI initiation and those who did not (*p* = 0.6878).

During the 12 months after ICI initiation, 691 (12.5%) patients developed CAEs. After adjusting for differences in follow-up, the rate was estimated to be 0.20 (95% CI: 0.19–0.22) per person/year. The most common CAEs were arrhythmia (9.3%) followed by myocarditis (2.1%), acute MI (1.7%), pericarditis (1.2%), and cardiomyopathy (0.9%). Estimated rates per person/year for each type of CAE are reported in Supplemental Table S4. Table 3 presents the aHRs estimated from competing risk regressions by first ICI agent(s) and by ICI class. The aHRs for covariates were very similar in both models. Here, we only report the results from the model by first ICI agent(s). Risk of CAEs increased with age. Patients aged 65–74 years (aHR: 1.45; 95% CI: 1.03–2.05; *p* = 0.035) and 75 years or older (aHR: 2.11; 95% CI: 1.47–3.03; *p* < 0.001) at the time of ICI initiation were statistically significantly more likely to develop CAEs within one year compared to patients aged 18–44 years. Males (aHR: 1.31; 95% CI: 1.12–1.54; *p* = 0.001) and blacks (aHR: 1.34; 95% CI: 1.02–1.77; *p* = 0.038) had higher risk of developing CAEs compared to females and whites, respectively. Patients with prior diagnosis of CHF (aHR: 2.01; 95% CI: 1.43–2.83; *p* < 0.001) and MI (aHR: 1.50; 95% CI: 1.04–2.17; *p* = 0.029) had a statistically higher risk of CAEs compared to those without those prior diagnoses. Having lung cancer statistically significantly increased (aHR: 1.24; 95% CI: 1.02–1.50; *p* = 0.032) and liver cancer significantly decreased (aHR: 0.45; 95% CI: 0.27–0.74; *p* = 0.002) the risk of CAEs compared to those with other cancers.

**Figure 2:**
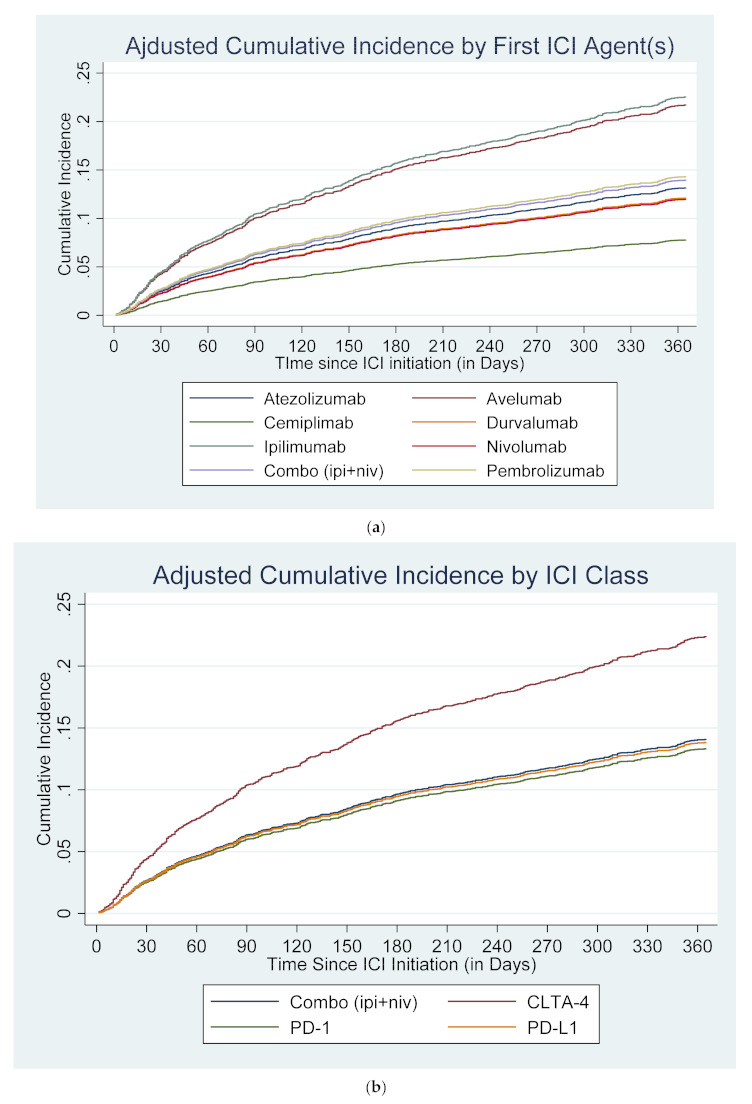
Cumulative incidence of cardiotoxicity after competing risk regressions, adjusted for covariates. (**a**) By first ICI agent(s), aHR (95% CI). Nivolumab: 1.00 (reference); atezolizumab: 1.11 (0.71–1.72), *p* = 0.65; avelumab: 1.92 (0.85–4.34), *p* = 0.12; cemiplimab: 0.64 (0.08–4.75), *p* = 0.66; durvalumab: 1.01 (0.47–2.16), *p* = 0.97; ipilimumab: 2.00 (1.49–2.70), *p* < 0.01; pembrolizumab: 1.21 (1.01–1.46), *p* = 0.04; combination (niv + ipi): 1.18 (0.85–1.64), *p* = 0.32. (**b**) By ICI Class, aHR (95% CI). PD-1: 1.00 (reference); CTLA4: 1.77 (1.34–2.34), *p* < 0.01; PD-L1: 1.04 (0.74–1.46), *p* = 0.82; combination (niv + ipi): 1.06 (0.78–1.45), *p* = 0.71. ICI: immune checkpoint inhibitor; CTLA-4: Cytotoxic T-lymphocyte associated-antigen-4; PD-1: programmed death receptor-1; PD-L1: programmed death-ligand 1; Combo (ipi+niv): combination of ipilimumab and nivolumab; aHR: adjusted hazard ratio; 95% CI: 95% confidence interval.

After adjusting for these differences, patients who initiated on monotherapy with ipilimumab (aHR: 2.00; 95% CI: 1.49–2.70; *p* < 0.001) or pembrolizumab (aHR: 1.21; 95% CI: 1.01–1.46; *p* = 0.040) had a higher risk of developing CAEs within one year compared to nivolumab monotherapy. Risk of cardiotoxicity was also elevated among patients who used avelumab as their first ICI treatment (aHR: 1.92; 95% CI: 0.85–4.34; *p* = 0.117), although the risk was not statistically significant likely due to the small sample size (n = 29). Similarly, when compared across ICI classes, CTLA4 inhibitors (ipilimumab) had the highest risk of CAEs compared to other classes (aHR: 1.77; 95% CI: 1.35–2.34; *p* < 0.001). The adjusted cumulative incidence rates by the first ICI agent(s) and by ICI class are presented in Figure 2a,b.

**Figure 3:**
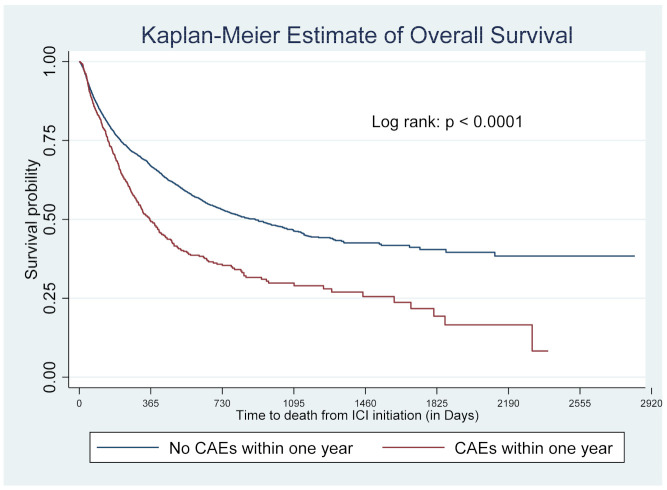
Kaplan–Meier estimate of overall survival. CAE: cardiac adverse event. ICI: immune checkpoint inhibitor.

**Figure 4:**
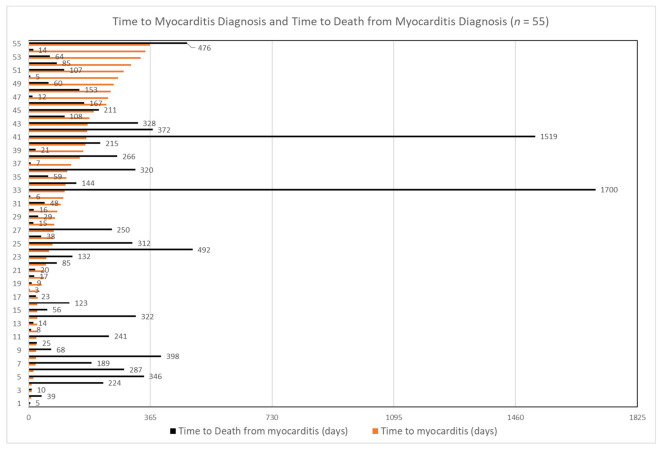
Time to myocarditis diagnosis and time to death from myocarditis diagnosis in patients who developed myocarditis and died during the study period (*n* = 55).

Risk of death was higher among patients who developed CAEs within one year of ICI initiation; 55% died during our study period compared to 35% in patients who did not. Figure 3 presents the survival curves accounting for censoring due to different follow-up periods. The median survival time was a little over a year after ICI initiation in patients who developed CAEs within one year of ICI initiation compared to about 2.5 years for those who did not develop CAEs (*p* < 0.0001) (Figure 3). Table 4 reports the aHRs from the time-varying Cox regression model. While the baseline HR was 1.46 (95% CI: 1.26–1.70; *p* < 0.001) between patients who developed CAEs within one year and those who did not, the HR widened by approximately 16% each year. (Table 4). Among the 116 patients who developed myocarditis within the first year (2.1%), the median time to myocarditis diagnosis was 115 days, with 25% developing it within 10 days and 75% developing it within 6 months of ICI initiation. Among them, 55 (47%) patients died afterward. While most of the deceased patients (49/55, 89.1%) died within one year after developing myocarditis, four (7%) died during the second year and only two patients lived beyond four years (3.6%) (Figure 4).

## 4. Discussion

In this large EMR-based database from multiple health care organizations, we determined the risk of cardiotoxicity within one year of ICI initiation among patients who had received at least one cycle of ICI for multiple cancers. Similar to other large population-based studies using real-world data, we found a higher incidence of cardiotoxicity than previously reported in clinical trials. By month 12, 12.5% developed cardiotoxicity. The most common cardiotoxicity was arrhythmia (9.3%) and 2.1% developed myocarditis by month 12. 

### 4.1. Cardiotoxicity 

Cardiotoxicity is often underreported in clinical trials. Hu et al. (2017) reviewed 22 clinical trials involving single-agent PD1 and PD-L1 inhibitors in non-small-cell lung cancer (NCSLC). Overall, cardiotoxicity was reported in 12 patients (one myocarditis, one pericardial effusion, one cardiac tamponade, one pulmonary embolism, one constrictive pericarditis, two MI, two cardiorespiratory arrest, and three cardiac (heart) failure) out of a combined 1784 patients across 10 trials that reported any cardiotoxicity (0.7%) [30]. 

Our estimate is higher than that reported in clinical trials but similar to other studies using real world databases. Using a US commercial insurance database (OptumLabs Data Warehouse, https://www.optum.com/about-us.html, accessed on 17 February 2022), Cathcart-Rake et al. (2020) evaluated irAEs incidence in 3164 patients (https://www.optum.com/about-us.html, accessed on 17 February 2022) with NCSLC who received PD-1 or PD-L1 inhibitors between 2015 and 2017 [22]. By month 9, 9.07% experienced an arrhythmia, 2.85% had acute MI, 0.89% had myocarditis, 1.65% had pericarditis, and 1.02% had cardiomyopathy [22]. A nationwide Danish study using data from 2011 to 2017 examined a composite outcome of cardiac events (arrhythmia, pericarditis, myocarditis, and heart failure) or cardiovascular death [31]. The one-year absolute risk of cardiac events after ICI initiation was 6.6% (95% CI: 1.8–11.3) in melanoma patients treated with PD-1 inhibitors, 7.5% (3.7–11.3) in melanoma patients treated with CTLA-4 inhibitors, and 9.7% in lung (95% CI: 6.8–12.5) cancer patients treated with PD-1 inhibitors [31]. Compared to patients without ICI treatment, the risk of cardiac events was higher in patients treated with ICIs but decreased after 6 months [31]. Chitturi et al. (2019) studied 252 patients with pathologically confirmed lung cancer who received ICIs between August 2015 and August 2018 from a single institution [32]. In this study, major adverse cardiac events, defined as a composite of cardiovascular death, nonfatal infarction, nonfatal stroke, and hospitalization for heart failure, occurred in 13.3% of patients during a median follow-up of 6 months with a median time to event of 51 days [32]. In another one-institution study of 424 cancer patients who received any ICI treatment from 2011 to 2017, 14.6% developed cardiovascular diseases after initiation of ICI treatment, defined as a new ICD diagnosis code for cardiomyopathy, heart failure, arrhythmia, heart block, pericardial disease, or myocarditis [33]. Similar to our study, the most frequently diagnosed cardiac condition was arrhythmia (6.1%) and 5.4% of patients had newly diagnosed heart failure [33]. However, the aforementioned studies used data of earlier years (mostly from 2017, [22,31,33]), from a single institution [32,33], or focusing on one or two cancer sites [22,31,32]. We provided updated information with newer data and a much larger cohort across all cancer sites and all seven FDA-approved ICIs and combination therapies. 

### 4.2. Overall Survival 

Mortality in ICI treated patients who developed cardiotoxicity was higher compared to those who did not. In our study, we found that 55% of patients who developed CAEs within one year of ICI initiation died compared to 35% in those who did not. This finding is consistent with previous studies. In a single-institution study of 424 cancer patients who received any ICI treatment from 2011 to 2017, 66.1% of patients with a concomitant diagnosis of incident cardiovascular disease died compared to 41.4% among those who did not (odds ratio (OR): 2.77; 95% CI: 1.55–4.95; *p*  =  0.0006) [33]. Escudier et al. reported a fatality rate of 27% among 30 patients with ICI-associated cardiotoxicity including left-ventricular systolic dysfunction, Takotsubo syndrome-like appearance, atrial fibrillation, ventricular arrhythmia, conduction abnormalities, and pericardial effusions [17]. Of the 122 ICI-associated myocarditis cases, 20 pericardial disease cases, and 82 vasculitis cases identified in the WHO’s global database of individual case safety reports, 50% of myocarditis cases, 21% of pericardial disease cases, and 6% of vasculitis cases resulted in death, respectively [13]. 

### 4.3. Risk Factors

Previous studies of clinical trial data have found elevated risk for severe cardiotoxicity when an ICI was used in combination (nivolumab plus ipilimumab) or with chemotherapy. For instance, Hu et al. (2021) conducted a meta-analysis of cardiac adverse events in 20,244 patients from 25 clinical trials involving monotherapy or combination therapy of ICIs plus chemotherapy published up to October 2020 [34]. Cardiac adverse events were classified into six major categories: arrhythmias, cardiac failure, coronary artery disease, pericardial disease, cardiac arrest, and myocardial disease [34]. Compared with nivolumab or ipilimumab monotherapy, combined nivolumab and ipilimumab therapy showed significant increases in grade 5 arrhythmias (OR: 3.90; 95% CI: 1.08–14.06) where arrhythmias was defined broadly to include atrial fibrillation, atrial flutter, atrial tachycardia, atrioventricular block, arrhythmia supraventricular, complete atrioventricular block, bradycardia, bifascicular block, sinus bradycardia, sinus tachycardia, supraventricular tachycardia, tachycardia, ventricular arrhythmia, ventricular tachycardia, and ventricular fibrillation [34]. In our study, use of the nivolumab plus ipilimumab combination as the first ICI treatment trended towards a higher risk of CAEs compared to nivolumab monotherapy but did not reach statistical significance after adjusting for differences in patient characteristics. On the other hand, patients initiated on ipilimumab, pembrolizumab, and avelumab monotherapies were found to have an elevated risk compared to nivolumab monotherapy, although avelumab did not achieve statistical significance due to small sample size. These results are not directly comparable to Hu et al. (2021) because the severity of cardiotoxicity could not be ascertained in our study. 

In our study, there were no differences in prior radiation and chemotherapy use between the CAEs group and no CAES group. Adjusting for prior use of these treatments or the chemotherapy agents did not estimate a statistically significant association with CAEs either. We therefore did not include them in the reported analysis. In the above meta-analysis by Hu et al. (2021) using clinical trial data, PD-1 inhibitor plus chemotherapy showed a significant increase in grade 1–5 myocardial disease (OR: 5.09; 95% CI: 1.11–23.32) compared with chemotherapy alone [34]. Compared with combined chemotherapy and nivolumab/ipilimumab, combined nivolumab and ipilimumab therapy showed a significant increase in grade 1–5 arrhythmias (OR: 2.49; 95% CI: 1.30–4.78) [34]. However, a study of ICI use in elderly patients with lung cancer using the SEER-Medicare database found that the ICI-plus-chemotherapy (used either concurrently or sequentially) group had equal or lower risk of cardiotoxicity, including acute coronary syndrome (HR: 0.82; 95% CI: 0.64–1.05; *p* = 0.10), heart failure (HR: 0.74; 95% CI: 0.62–0.88; *p* = 0.0007), cardiac arrhythmia (HR: 0.72; 95% CI: 0.63–0.82; *p* < 0.0001), and heart blocks (HR: 0.48; 95% CI: 0.30–0.76; *p* < 0.0001), compared to the traditional chemotherapy treatment only group [35]. Given these contracting results, future studies are warranted to further investigate this in large population-based real world databases. 

We also found older age, male gender, black race, and history of CHF and MI were associated with increased risk of developing new CAEs within one year of ICI initiation. In the general population, risk of cardiovascular diseases (CVD) increases with age and is higher among blacks compared to other races [36]. Incidence of CVD is also lower in women than in men, although women have a higher mortality and worse prognosis after acute cardiovascular events [37]. Previous literature found increased risk of ICI-induced cardiotoxicity in males [15]. However, the findings are inconclusive regarding whether patients with pre-existing cardiovascular diseases are at increased risk of ICI-induced cardiotoxicity [1]. On the other hand, the occurrence of irAEs is often an indicator of ICI activity [38]. In a comprehensive review of existing evidence on the involvement of sociological factors, lifestyles, and metabolic disorders in modulating the ICI response in cancer patients, Deshpande et al. (2020) reported evidence on direct or indirect links of age, sex, race, lifestyle factors (diet, exercise, alcohol, and smoking), obesity, and psycho-emotional stress with ICI response; however, the findings on the selective benefits of ICI by patient’s sex or race are conflicting [39]. These findings and ours underscore the need for consideration of these factors when prescribing ICIs. 

### 4.4. Myocarditis

The data on ICI-associated cardiotoxicities focus mostly on the development of myocarditis [40]. While clinical trials have reported a low rate of myocarditis (0.09%), real-world data have reported a higher rate. In a one-institution study of 964 patients treated with ICI from 2013 to 2017, 11 (1.14%) patients developed ICI-associated myocarditis [12]. Cathcart-Rake (2020) evaluated irAEs incidence in patients with NSCLC who received PD-1 or PD-L1 inhibitors using a US commercial insurance database and found the rate of myocarditis to be 0.89% by month 9 after ICI initiation [22]. Using the same set of diagnosis codes for cardiac irAEs, we found that 2.1% developed myocarditis within one year after ICI initiation across multiple cancers and all seven FDA-approved ICIs and ICI combination therapies. 

Although rare, myocarditis is often fatal. In the 18 patients who developed severe myocarditis from the Bristol Myers Squibb corporate safety database, six (33%) died [11]. Patients treated with a combination of nivolumab and ipilimumab experienced a higher incidence of myocarditis (0.27% vs. 0.06%) and fatality rate compared to nivolumab monotherapy (5/8, 63% vs. 1/10, 10%) [11]. In our study, among the 116 patients who developed myocarditis within one year, 47% died afterwards with nearly 89% of them having died within one year of myocarditis diagnosis. In 122 cases of ICI-associated myocarditis identified from the WHO’s global adverse report database of individual case safety reports, death occurred in 50% of the cases [13]. 

Despite the known risk, ICI-induced myocarditis is still poorly understood. Pathological studies have demonstrated heart injury from T-cell infiltration within myocardium with or without myocyte degeneration and necrosis of non-ischemia origin [1,41]. A recent study by Power et al. (2021) reported electrocardiographic and arrhythmogenic features of ICI-myocarditis among 125 patients identified from an online registry of 49 institutions and 11 countries [42]. The results from this study establish ICI-myocarditis to be highly arrhythmogenic and define specific electrocardiographic features that will help clinicians diagnose and prognosticate the syndrome [42]. A wide range of ECG abnormalities have been presented, including conduction blocks, decreased voltage, and repolarization abnormalities that frequently degenerate to malignant arrhythmias [42]. Currently, endomyocardial biopsy remains the gold standard for confirming myocarditis, but it is rarely performed in clinical practice due to its invasive nature. In our study and others [33], arrhythmias were found to be the most commonly diagnosed CAEs, some of which could be due to undiagnosed underlying myocarditis. 

Although ICI-associated myocarditis typically occurs after 2–3 ICI cycles, a wide range of onset times have been reported, from 2 to 454 days after starting ICI treatment [1,11,12,17,43]. Our study findings are consistent with these reports. Among the 116 patients who developed myocarditis within one year, the median time to onset was 115 days with 25% developing within 10 days and 75% within 6 months. Some late-onset of ICI-associated myocarditis occurred more than a year after starting ICI therapy [44], although it is unclear whether it was due to delayed development of myocarditis or resulted from myocarditis that began much earlier, or was caused by cumulative injury to the heart due to persistent systemic immune activation and inflammation [1]. Occupation of PD-1 and PD-L1 receptors may remain long after the infusions of ICI have stopped, which may partially explain the wide range in the median time to onset of myocarditis and requires clinicians to remain vigilant when patients present with myocarditis-like symptoms late after starting ICI or who are no longer being actively treated with an ICI [1]. 

### 4.5. Cardiotoxicity by ICI Agents 

Most studies of cardiotoxicity focused on earlier ICIs such as ipilimumab, nivolumab, pembrolizumab, or their combinations. Use of ICI combination therapy increases the risk of myocarditis compared to monotherapy [11]. Few studies have reported on cardiotoxicity associated with the newer ICIs, most of which are PD-L1 inhibitors. It was speculated that PD-L1 inhibitors may be associated with lower adverse events because they still allow for the interaction of PD-1 with its other ligand PD-L2 [45]. However, a systematic review of published data of trials utilizing PD-1 (nivolumab and pembrolizumab) and PD-L1 inhibitors (atezolizumab, durvalumab, and avelumab) in NSCLC patients found similar toxicity between PD-1 and PD-L1 inhibitors [45]. A significantly higher (but not reaching statistical significance) rate of toxicity was observed in durvalumab compared to other ICIs (75% vs. 62–67%), which warrants future studies [45]. Cardiotoxicity was not separately studied in this study [45]. In our study, we found that after adjusting for patients’ demographic and clinical characteristics, patients initiated on ipilimumab and pembrolizumab monotherapies had a statistically significant higher risk of developing CAEs within one year after ICI initiation compared to patients initiated on nivolumab monotherapy, with the ipilimumab group having nearly double the hazard of CAEs compared to the nivolumab group. Patients initiated on avelumab monotherapy also were estimated as having a highly elevated risk but the effect was not statistically significant due to the small sample size. Larger studies are needed to confirm this finding. We did not find statistically significant differences in CAE risk between other PD-L1 agents (atezolizumab and durvalumab) and nivolumab monotherapy, nor did we find statistically significant difference by class between PD-L1 and PD-1 inhibitors when comparing patients’ initial ICI treatment. 

### 4.6. Cardiotoxicity by Type of Cancer

Few studies have compared ICI-associated cardiotoxicity across cancer sites. In a recently published nationwide Danish study using data from 2011 to 2017, patients with incident lung cancer or melanoma were studied [31]. Cardiotoxicity was defined as a composite outcome of cardiac events (arrhythmia, pericarditis, myocarditis, or heart failure) or cardiovascular death. The one-year absolute risk of cardiac events was 6.6% (95% CI: 1.8–11.3) in melanoma patients treated with PD-1 inhibitors, 7.5% (3.7–11.3) in melanoma patients treated with CTLA-4 inhibitors, and 9.7% in lung (95% CI: 6.8–12.5) cancer patients treated with PD-1 inhibitors [31]. In addition, lung cancer and melanoma patients were studied separately; thus, it is unknown whether these differences were statistically significant [31]. Moreover, higher proportions of lung cancer patients have pre-existing cardiovascular conditions prior to ICI initiation compared to melanoma patients [31]. In the adjusted analysis after adjusting for differences in patient characteristics, melanoma patients with ICIs (vs. melanoma patients without ICIs) were estimated to have a much higher HR of cardiac events compared to lung cancer patients with ICIs (vs. lung cancer patients without ICIs) (e.g., for risk of cardiac events occurring <6 months after ICI initiation: melanoma with PD-1 inhibitor (HR: 4.30; 95% CI: 1.38–13.42), lung cancer with PD-1 inhibitor (HR: 2.14; 95% CI: 1.50–3.05)) [31]. However, these HRs could not be directly compared between melanoma and lung cancer patients because the reference groups were different (melanoma patients without ICIs and lung cancer patients without ICIs) [31]. Waheed et al. compared the primary cancer diagnosis between patients with newly diagnosed cardiovascular disease and those without and find no statistically significant difference; no further adjusted analyses by cancer sites were conducted [33]. Two studied only lung cancer patients [22,32]. These studies used different designs and definitions of cardiotoxicity and could not be compared directly [22,31,32,33]. 

In our study, we included ICI users with malignant cancers in multiple sites and found that having lung cancer independently increased the risk of ICI-associated cardiotoxicity compared to those without lung cancer. Prior thoracic radiation treatment for lung cancer can cause injury to the heart. Animal studies have characterized radiation-induced heart disease with fibrosis and acute production of inflammatory cytokines, which can compound ICI-induced cardiac dysfunction and cause cumulative cardiotoxicity [46]. Interestingly, in a recent review study of 134 published cardiotoxicity cases, lung cancer appeared to have a longer time of onset of cardiotoxicity comparing to other cancer sites [18]. The reason for this difference is unknown and should be further investigated in future larger studies. We also found liver cancer to be associated with statistically significantly lower risk of ICI-associated cardiotoxicity compared to other cancer sites. To the best of our knowledge, no studies have compared ICI-associated cardiotoxicity between liver cancer and other cancers. The reason for this reduced risk is unknown and warrants future studies. In a large study of health insurance claims database, Wang et al. found that although ICIs were associated with increased risk of developing irAEs in patients with all seven cancer types under study compared to chemotherapy, the risk varied across the cancer types; however, cardiac irAEs were not included in the analysis [47]. 

Although ICIs were initially approved as salvage treatments when patients failed other treatments, they are increasingly being approved for earlier stage cancers and in adjuvant settings. These patients may survive longer and are at more risk of late-onset ICI-associated cardiotoxicity; thus, continued vigilance is needed. 

### 4.7. Other irAEs

The most common irAEs are endocrine AEs, with 44.9% patients experiencing those AEs in the first year after ICI initiation. This estimate is consistent with previous studies. A review of phase-III studies of ICIs found varying estimates ranging from 3.8% for nivolumab in one study to up to 30% in nivolumab and ipilimumab combinations for endocrine AEs of any grade; however, high-grade (grade 3–4) endocrine AEs were rare (0–5.5% across studies) [48]. AKI was also high in our study with 22.1% of patients having developed AKI within one year of ICI initiation. AKI occurred in 35.3% of patients who developed CAEs within one year of ICI initiation and 20.2% in those who did not. The difference was statistically significant (*p* < 0.0001). Because the focus of this study was on cardiac AEs, we did not exclude patients with pre-existing AKIs or renal disease. In this study, 11.2% had pre-existing renal disease. The proportion of patients with pre-existing renal disease was higher in patients who developed CAEs within one year of ICI initiation than those who did not (13.7% vs. 10.8%, *p* = 0.0221). We adjusted for this difference in the regression analysis. In a study of lung cancer patients, Cathcart-Rake et al. (2020) excluded patients with pre-existing codes for AKI and other irAEs, and the estimated incidence rate of AKI by month 9 was 7.33% (95% CI: 6.18, 8,69) [22]. A review of published phase-2 and -3 clinical trials found the overall incidence of AKI to be 2–5%, with high-grade AKI (grade 2 or 3) needing dialysis to be 0.6% [49]. However, other studies using routine practice data have reported higher incidences of 13.9% [50] to 29% [51], varying by ICI agent and higher in the combination therapy [52].

### 4.8. Limitations

This is a retrospective study using real world data from a network of multiple health care systems. The major advantages are its large sample size (5518 patients), newer data, and its covering of multiple cancer sites and all seven FDA-approved agents, although the number of records for newer agents (atezolizumab, avelumab, cemiplimab, and durvalumab) are relatively small (21 to 201 patients depending on the agent). A major limitation is that tumor-specific characteristics such as stage and histology were not included due to low reporting to the network. Moreover, severity of cardiotoxicity could not be determined. ICI-related cardiotoxicity was defined based on diagnosis codes, which may be subject to inaccurate reporting. To reduce the risk of misclassification due to pre-existing conditions, only new diagnoses were considered and patients who had a relevant diagnose code from the list of CAEs before ICI initiation were excluded. We relied on this temporal relationship (not present before ICI initiation but present within one year after ICI initiation) to establish incident cases, which is a common epidemiology study design. We further restricted the study to only cases discovered within one year of ICI initiation to reduce the chances of these events occurring later due to the natural aging process, other drugs, or newly developed conditions unrelated to ICI use. Nonetheless, it is possible that some cases may have been misclassified and late-onset cases may have been missed due to this cutoff of 12 months. Moreover, cardiotoxicity was limited to the list of CAEs examined. Recent studies have found associations between ICIs and increased thromboembolic events [53] and atherosclerotic plaque [40]. Therefore, we may have underestimated the incidence of ICI-associated cardiotoxicity.

Thoracic radiation therapy has been shown to increase the risk of cardiotoxicity due to its proximity to the heart. However, radiation fields could not be determined from the procedure codes. Nonetheless, the analysis adjusted for lung cancer diagnosis, which is the major site for ICI indication and thoracic radiation. Other cancer sites such as breast and esophagus may also receive thoracic radiation. However, by October 2019, only atezolizumab had been approved for triple-negative breast cancer (March 2019) and pembrolizumab in combination with chemotherapy was approved for esophageal cancer as a third or subsequent line of treatment (September 2017) [2]. The use of ICIs for patients with these types of cancer was very low during our study period and, therefore, was not separately analyzed (Table 1). As a retrospective study, it is possible that unmeasured residual confounders such as diet, physical activity, and family history may influence the association between ICI use and CAEs [39]. We also did not include smoking status and BMIs because not all HCOs reported this information to TriNetX. However, previous studies that included smoking status and/or BMI did not find a significant effect of either variable on ICI-associated cardiotoxicity [40,48]. Information on tumor PD-L1 proportion and mutation burden are very incomplete and, therefore, were not used in the analysis. Although both affect responses to ICIs, it is unknown whether they affect the risk of ICI-associated cardiotoxicity. We also did not assess the association of biomarkers with ICI-associated cardiotoxicity due to incomplete information. Biomarkers such as troponin and B-type natriuretic peptide are often elevated in patients with ICI-associated myocarditis [12] and can aid in diagnosis and assessing prognosis [54]. However, it is still unclear whether these biomarkers could be used to identify high-risk patients who will develop myocarditis [55].

## 5. Conclusions

In this large EMR-based database from multiple health systems, we estimated the incidence rates of CAEs within one year after ICI initiation. While there were no differences in risk of cardiotoxicity between PD-1 and PD-L1 inhibitors overall, ipilimumab and pembrolizumab use may increase the risk of cardiotoxicity compared to other agents and should be closely monitored in the future, especially with the rapidly expanded use of pembrolizumab. Avelumab was also estimated as having a highly elevated risk compared to nivolumab and other PD-L1 agents, although the estimate did not reach statistical significance. Given that little is known on the cardiotoxicity among PD-L1 agents or avelumab, future larger studies are urgently needed to confirm this finding.

## Figures and Tables

**Table 1:** Patient Characteristics.

Variables	Cardiotoxicity Developed within One Year after ICI Initiation						%
	Yes (*n* = 691)		No (*n* = 4827)		*p*-Value	Total (*n* = 5518)	
	*n*	%	*n*	%	Chi-Square	*n*	
**Age at ICI initiation**					<0.0001		
18–44	**42**	6.1%	**470**	9.7%		**512**	9.3%
45–54	**85**	12.3%	**647**	13.4%		**732**	13.3%
55–64	**187**	27.1%	**1414**	29.3%		**1601**	29.0%
65–74	**212**	30.7%	**1407**	29.1%		**1619**	29.3%
≥75	**165**	23.9%	**719**	14.9%		**884**	16.0%
**Sex**					0.0012		
F	**254**	36.8%	**2088**	43.3%		**2342**	42.4%
M	**437**	63.2%	**2739**	56.7%		**3176**	57.6%
**Hispanic**					0.0144		
Yes	**10**	1.4%	**165**	3.4%		**175**	3.2%
No	**647**	93.6%	**4389**	90.9%		**5036**	91.3%
Unknown	**34**	4.9%	**273**	5.7%		**307**	5.6%
**Race**					0.0718		
American Indian or Alaska Native	**1**	0.1%	**10**	0.2%		**11**	0.2%
Asian	**9**	1.3%	**63**	1.3%		**72**	1.3%
Black or African American	**60**	8.7%	**322**	6.7%		**382**	6.9%
Native Hawaiian/Other Pacific Islander	**2**	0.3%	**4**	0.1%		**6**	0.1%
Unknown	**20**	2.9%	**223**	4.6%		**243**	4.4%
White	**599**	86.7%	**4205**	87.1%		**4804**	87.1%
**Charlson Comorbidity Index ^1^**							
0	**230**	33.3%	**1874**	38.8%	0.0042	**2104**	38.1%
1	**171**	24.7%	**1245**	25.8%		**1416**	25.7%
2	**121**	17.5%	**760**	15.7%		**881**	16.0%
≥3	**169**	24.5%	**948**	19.6%		**1117**	20.2%
**Comorbidities** ^1^							
AIDS/HIV	**5**	0.7%	**28**	0.6%	0.6472	**33**	0.6%
Cerebrovascular disease	**89**	12.9%	**496**	10.3%	0.0375	**585**	10.6%
Congestive heart failure	**41**	5.9%	**124**	2.6%	<0.0001	**165**	3.0%
Chronic pulmonary disease	**201**	29.1%	**1287**	26.7%	0.1790	**1488**	27.0%
Dementia	**5**	0.7%	**26**	0.5%	0.5429	**31**	0.6%
Diabetes without chronic complication	**73**	10.6%	**465**	9.6%	0.4403	**538**	9.7%
Diabetes with chronic complication	**47**	6.8%	**259**	5.4%	0.1229	**306**	5.5%
Diabetes secondary to drug use	**9**	1.3%	**53**	1.1%	0.6334	**62**	1.1%
Any diabetes ^2^	**120**	17.4%	**733**	15.2%	0.1380	**853**	15.5%
Hemiplegia/paraplegia	**17**	2.5%	**97**	2.0%	0.4360	**114**	2.1%
Metastatic solid tumor	**599**	86.7%	**4141**	85.8%	0.5260	**4740**	85.9%
Myocardial infarction	**33**	4.8%	**113**	2.3%	0.0002	**146**	2.6%
Mild liver disease	**146**	21.1%	**1024**	21.2%	0.9591	**1170**	21.2%
Peptic ulcer disease	**21**	3.0%	**122**	2.5%	0.4285	**143**	2.6%
Peripheral vascular disease	**108**	15.6%	**592**	12.3%	0.0129	**700**	12.7%
Renal disease	**95**	13.7%	**522**	10.8%	0.0221	**617**	11.2%
Rheumatic disease	**15**	2.2%	**127**	2.6%	0.4748	**142**	2.6%
Moderate/severe liver disease	**11**	1.6%	**138**	2.9%	0.0546	**149**	2.7%
Hypertension	**351**	50.8%	**2175**	45.1%	0.0046	**2526**	45.8%
**Primary Cancer Site ^3^**							
Esophageal	**13**	1.9%	**87**	1.8%	0.8843	**100**	1.8%
Gastric	**12**	1.7%	**118**	2.4%	0.2511	**130**	2.4%
Colon	**21**	3.0%	**202**	4.2%	0.1526	**223**	4.0%
Liver	**19**	2.8%	**317**	6.6%	<0.0001	**336**	6.1%
Hodgkin’s	**10**	1.5%	**41**	0.9%	0.1246	**51**	0.9%
Melanoma	**226**	32.7%	**1635**	33.9%	0.5444	**1861**	33.7%
Lung	**292**	42.3%	**1676**	34.7%	<0.0001	**1968**	35.7%
Renal cell carcinoma	**54**	7.8%	**413**	8.6%	0.5126	**467**	8.5%
Urothelial (urethra/bladder/ureter)	**43**	6.2%	**296**	6.1%	0.9260	**339**	6.1%
Markel cell carcinoma	**7**	1.0%	**28**	0.6%	0.1800	**35**	0.6%
Head and neck	**46**	6.7%	**364**	7.5%	0.4073	**410**	7.4%
Breast	**40**	5.8%	**214**	4.4%	0.1118	**254**	4.6%
Cervix	**3**	0.4%	**51**	1.1%	0.1201	**54**	1.0%
Primary mediastinal (thymic) large B-cell lymphoma	**1**	0.1%	**1**	0.02%	0.1092	**2**	0.04%
Other	**47**	6.8%	**404**	8.4%	0.3011	**451**	8.2%
**Prior Cancer Treatment**							
**Radiation (Y/N)**	**254**	36.8%	**1620**	33.6%	0.0970	**1874**	34.0%
**Chemotherapy (Y/N)**	**150**	21.7%	**1073**	22.2%	0.7576	**1223**	22.2%
Alkylating agent	**131**	19.0%	**896**	18.6%	0.8026	**1027**	18.6%
Antimetabolite	**73**	10.6%	**506**	10.5%	0.9477	**579**	10.5%
Antimitotic agent	**60**	8.7%	**393**	8.1%	0.6278	**453**	8.2%
Antitumor antibiotic	**26**	3.8%	**175**	3.6%	0.8571	**201**	3.6%
Enzyme inhibitor	**0**	0.0%	**8**	0.2%	0.2842	**8**	0.1%
Plant alkaloid	**19**	2.7%	**147**	3.0%	0.6704	**166**	3.0%
Proteasome inhibitor	**0**	0.0%	**10**	0.2%	0.2311	**10**	0.2%
Topoisomerase I inhibitor	**6**	0.9%	**66**	1.4%	0.2797	**72**	1.3%
Chemotherapy—nonspecific	**5**	0.7%	**33**	0.7%	0.9055	**38**	0.7%
**Other irAEs developed within one year of ICI initiation ^4^**							
Hematologic	**381**	55.1%	**2013**	41.7%	<0.0001	**2394**	43.4%
Pulmonary (pneumonitis)	**277**	40.1%	**1071**	22.2%	<0.0001	**1348**	24.4%
Endocrine	**352**	50.9%	**2126**	44.0%	0.0007	**2478**	44.9%
Renal (acute kidney injury)	**244**	35.3%	**974**	20.2%	<0.0001	**1218**	22.1%
Neurological	**234**	33.9%	**1200**	24.9%	<0.0001	**1434**	26.0%
Hepatic	**180**	26.0%	**913**	18.9%	<0.0001	**1093**	19.8%
Gastrointestinal	**197**	28.5%	**1075**	22.3%	0.0003	**1272**	23.1%
Skin (vitiligo)	**59**	8.5%	**360**	7.5%	0.3160	**419**	7.6%

ICI: immune checkpoint inhibitor; irAE: immune-related adverse event. ^1^ Comorbidities and Charlson Comorbidity Index (CCI) values were calculated based on diagnoses before or on the day of ICI initiation. ICD 9 and 10 codes used to calculate the CCI were from Quan et al. (2005). Primary cancer diagnoses were not included in the calculation of the index but metastatic/secondary cancers were included. Hierarchy coding was applied to prevent duplicated accounting. For instance, if a person had diagnoses of both a mild and a severe form of the disease (e.g., mild and moderate/severe liver disease, or diabetes with and without chronic complications), the patient was only scored on the more severe disease in the CCI. ^2^ Any diabetes included patients who had any of the following diagnoses, diabetes without chronic complication, diabetes with chronic complication, or diabetes secondary to drug use, prior to or on the day of ICI initiation. ^3^ Primary cancer sites are based on diagnoses of primary cancers (excluding secondary diagnoses) before ICI initiation or 30 days after. Site variables are not mutually exclusive and patients may have multiple cancer sites. ^4^ Other irAEs included hematologic (anemia, thrombocytopenia, leukopenia), pneumonitis, endocrine (hypothyroidism, hyperthyroidism, hypophysitis/PGA, hyper/hypoparathyroidism, diabetes type I, dysfunctional uterine bleeding/infertility), renal (acute kidney injury/AKI), neurological (encephalitis/myelitis/encephalomyelitis, neuritis, meningitis), hepatic (hepatitis), gastrointestinal (GI) (colitis, pancreatitis, mucositis), and skin (vitiligo)).

**Table 2:** First immune checkpoint inhibitor treatments.

Variables		Cardiotoxicity Developed within One Year after ICI Initiation						%
		Yes (*n* = 691)		No (*n* = 4827)		*p*-Value	Total (*n* = 5518)	
		*n*	%	*n*	%		*n*	
**First ICI**	**Class**					0.0038		
Atezolizumab	PD-L1	**23**	3.3%	**178**	3.7%		**201**	3.6%
Avelumab	PD-L1	**7**	1.0%	**22**	0.5%		**29**	0.5%
Durvalumab	PD-L1	**7**	1.0%	**49**	1.0%		**56**	1.0%
Ipilimumab	CTLA-4	**85**	12.3%	**385**	8.0%		**470**	8.5%
Nivolumab	PD-1	**196**	28.4%	**1570**	32.5%		**1766**	32.0%
Pembrolizumab	PD-1	**324**	46.9%	**2181**	45.2%		**2505**	45.4%
Cemiplimab	PD-1	**1**	0.1%	**20**	0.4%		**21**	0.4%
Combo	Combo	**48**	6.9%	**422**	8.7%		**470**	8.5%
Nivolumab + Ipilimumab		**48**	6.9%	**420**	8.7%		**468**	8.5%
Pembrolizumab + ipilimumab		**0**	0.0%	**1**	0.0%		**1**	0.02%
Pembrolizumab + Nivolumab		**0**	0.0%	**1**	0.0%		**1**	0.02%
		**691**		**4827**				
**Number of different ICIs used (including agents used in combination)**						0.6878		
1		**587**	84.9%	**4043**	83.8%		**4630**	83.9%
2		**93**	13.5%	**692**	14.3%		**785**	14.2%
3		**11**	1.6%	**92**	1.9%		**103**	1.9%

ICI: immune checkpoint inhibitor. CTLA-4: Cytotoxic T-lymphocyte associated-antigen-4. PD-1: programmed death receptor-1. PD-L1: programmed death-ligand 1. Combo: combination.

**Table 3:** Hazard ratios for risk of cardiotoxicity estimated from competing risk regressions.

Variables	aHR	95% CI		*p*-Value		aHR	95% CI		*p*-Value
First ICI Treatment (ref: Nivolumab)					ICI Class (ref: PD-1)				
Atezolizumab	1.11	0.71	1.72	0.655	CTLA4	1.77	1.34	2.34	0.000
Avelumab	1.92	0.85	4.34	0.117	PD-L1	1.06	0.78	1.45	0.710
Cemiplimab	0.64	0.08	4.75	0.658	Combo (niv + ipi)	1.04	0.74	1.46	0.817
Durvalumab	1.01	0.47	2.16	0.974					
Ipilimumab	2.00	1.49	2.70	0.000					
Pembrolizumab	1.21	1.01	1.46	0.040					
Combo (niv + ipi)	1.18	0.85	1.64	0.324					
**Age at ICI initiation (ref = 18–44 years)**					**Age at ICI initiation** **(ref = 18–44 years)**				
45–54 years	1.34	0.92	1.95	0.124	45–54 years	1.34	0.92	1.94	0.128
55–64 years	1.30	0.92	1.83	0.139	55–64 years	1.30	0.92	1.83	0.139
65–74 years	1.45	1.03	2.05	0.035	65–74 years	1.44	1.02	2.04	0.037
75 years or older	2.11	1.47	3.03	0.000	75 years or older	2.11	1.47	3.03	0.000
**Sex (ref = Female)**					**Sex (ref = Female)**				
**Male**	1.31	1.12	1.54	0.001	**Male**	1.32	1.12	1.54	0.001
**Race (ref = White)**					**Race (ref = White)**				
Black	1.34	1.02	1.77	0.038	Black	1.34	1.01	1.76	0.040
Other	1.21	0.70	2.08	0.493	Other	1.22	0.71	2.10	0.471
Unknown	0.95	0.57	1.58	0.837	Unknown	0.93	0.56	1.55	0.777
**Hispanic (ref = No)**	0.53	0.25	1.10	0.088	**Hispanic (ref = No)**	0.53	0.26	1.1	0.092
**Charlson Comorbidity Index (ref = 0) ^1^**					**Charlson Comorbidity Index (ref = 0) ^1^**				
1	1.06	0.86	1.30	0.571	1	1.07	0.87	1.31	0.547
2	1.12	0.88	1.44	0.351	2	1.13	0.88	1.45	0.336
≥3	1.23	0.90	1.69	0.194	≥3	1.24	0.90	1.69	0.186
**Pre-existing Cardiovascular Diseases**					**Pre-existing Cardiovascular Diseases**				
Hypertension (ref = No)	1.03	0.87	1.23	0.704	Hypertension (ref = No)	1.03	0.87	1.23	0.711
Cerebrovascular disease (ref = No)	1.04	0.82	1.33	0.745	Cerebrovascular disease (ref = No)	1.04	0.82	1.33	0.737
Congestive heart failure (ref = No)	2.01	1.43	2.83	0.000	Congestive heart failure (ref = No)	2.00	1.43	2.81	0.000
Myocardial infarction (ref = No)	1.50	1.04	2.17	0.029	Myocardial infarction (ref = No)	1.51	1.04	2.18	0.028
Peripheral vascular disease (ref = No)	1.00	0.79	1.28	0.971	Peripheral vascular disease (ref = No)	1.00	0.79	1.28	0.968
**Renal disease (ref = No)**	1.01	0.75	1.36	0.950	**Renal disease (ref = No)**	1.01	0.75	1.35	0.960
**Moderate/severe liver disease (ref = No)**	0.81	0.41	1.57	0.527	**Moderate/severe liver disease (ref = No)**	0.82	0.42	1.60	0.553
**Primary Cancer Site ^2^**					**Primary Cancer Site ^2^**				
Lung (ref = No)	1.24	1.02	1.50	0.032	Lung (ref = No)	1.22	1.00	1.48	0.046
Melanoma (ref = No)	0.81	0.64	1.02	0.073	Melanoma (ref = No)	0.81	0.64	1.02	0.074
Renal cell carcinoma (ref = No)	0.89	0.64	1.23	0.481	Renal cell carcinoma (ref = No)	0.84	0.61	1.15	0.264
Urothelial (ref = No)	0.87	0.62	1.23	0.435	Urothelial (ref = No)	0.88	0.63	1.25	0.482
Head and neck (ref = No)	0.87	0.64	1.19	0.398	Head and neck (ref = No)	0.87	0.64	1.19	0.393
Meckel cell carcinoma (ref = No)	1.17	0.54	2.55	0.683	Meckel cell carcinoma (ref = No)	1.43	0.70	2.93	0.330
Liver (ref = No)	0.45	0.27	0.74	0.002	Liver (ref = No)	0.44	0.27	0.72	0.001

ICI: immune checkpoint inhibitor; aHR: adjusted hazard ratio; 95% CI: 95% confidence interval; CTLA-4: Cytotoxic T-lymphocyte associated-antigen-4; PD-1: programmed death receptor-1; PD-L1: programmed death-ligand 1; Combo (ipi+niv): combination of ipilimumab and nivolumab. ^1^ Charlson Comorbidity Index (CCI) values were calculated based on diagnoses before or on the day of ICI initiation. The ICD 9 and 10 codes used to calculate the CCI were from Quan et al. (2005). ^2^ Primary cancer sites are based on diagnoses of primary cancers (excluding secondary diagnoses) before ICI initiation or 30 days after. Site variables are not mutually exclusive and patients may have multiple cancer sites.

**Table 4:** Hazard ratios for overall survival.

Variables	aHR	95% CI		*p*-Value
**CAEs within One Year after ICI Initiation (ref = No)**	1.46	1.26	1.70	0.000
**Age at ICI Initiation (ref = 18–44 years)**				
45–54 years	0.94	0.78	1.14	0.540
55–64 years	0.95	0.80	1.12	0.537
65–74 years	0.96	0.81	1.14	0.631
75 years or older	1.00	0.83	1.21	0.995
**Sex (ref = Female)**				
Male	0.99	0.91	1.08	0.839
**Race (ref = White)**				
Black	0.69	0.57	0.84	0.000
Other	0.82	0.57	1.19	0.299
Unknown	1.11	0.86	1.43	0.436
**Hispanic (ref = No)**	1.04	0.78	1.39	0.778
**Charlson Comorbidity Index (ref = 0) ^1^**				
1	1.40	1.25	1.57	0.000
2	1.46	1.27	1.69	0.000
≥3	1.40	1.16	1.68	0.000
**Pre-existing Cardiovascular Diseases**				
Hypertension (ref = No)	0.96	0.87	1.05	0.356
Cerebrovascular disease (ref = No)	1.05	0.90	1.22	0.553
Congestive heart failure (ref = No)	0.99	0.76	1.28	0.911
Myocardial infarction (ref = No)	1.11	0.85	1.44	0.440
Peripheral vascular disease (ref = No)	0.90	0.78	1.03	0.134
**Renal disease (ref = No)**	0.89	0.75	1.06	0.206
**Moderate/severe liver disease (ref = No)**	1.32	0.98	1.77	0.064
**Primary Cancer Site ^2^**				
Lung (ref = No)	1.00	0.90	1.11	0.972
Melanoma (ref = No)	0.48	0.43	0.55	0.000
Renal cell carcinoma (ref = No)	0.78	0.66	0.93	0.006
urothelial (ref = No)	1.25	1.05	1.49	0.013
Head and neck (ref = No)	1.08	0.92	1.27	0.358
Meckel cell carcinoma (ref = No)	0.56	0.32	1.00	0.052
Liver (ref = No)	1.28	1.05	1.56	0.013
**Time-Varying Covariate**				
CAE × t	1.16	1.00	1.34	0.047

ICI: immune checkpoint inhibitor; aHR: adjusted hazard ratio; 95% CI: 95% confidence interval. ^1^ Charlson Comorbidity Index (CCI) values were calculated based on diagnoses before or on the day of ICI initiation. The ICD 9 and 10 codes used to calculate the CCI were from Quan et al. (2005). ^2^ Primary cancer sites are based on diagnoses of primary cancers (excluding secondary diagnoses) before ICI initiation or 30 days after. Site variables are not mutually exclusive and patients may have multiple cancer sites.

## Data Availability

Data are proprietary and cannot be shared.

## Supplementary Materials

The following supporting information can be downloaded at: https://www.mdpi.com/article/10.3390/cancers14051145/s1, Table S1. Procedure codes for ICIs; Table S2. Patient Characteristics by First ICI treatment; Table S3 Patient Characteristics by Primary Cancer Site; Table S4. Estimated Rate Per Person-Year.
