# Supplementary material for: Immune Checkpoint Inhibitors—Associated Cardiotoxicity
Source: Cancers (Basel). 2022 Feb 23;14(5):1145. doi: 10.3390/cancers14051145 (PMC8909315; doi:10.3390/cancers14051145)
Supplement: Supplementary file 1 [file cancers-14-01145-s001.zip › cancers-1547580-supplementary.pdf]

Table S1. Procedure codes for Immune Checkpoint Inhibitors (ICIs)

**ICI procedure codes**		
HCPCS	C9027	Injection, pembrolizumab, 1 mg (deprecated 2015)
HCPCS	C9453	Injection, nivolumab, 1 mg (deprecated 2015)
HCPCS	C9483	Injection, atezolizumab, 10 mg (deprecated 2017)
HCPCS	C9491	Injection, avelumab, 10 mg (deprecated 2017)
HCPCS	C9492	Injection, durvalumab, 10 mg (deprecated 2018)
HCPCS	J9022	Injection, atezolizumab, 10 mg
HCPCS	J9023	Injection, avelumab, 10 mg
HCPCS	J9228	Injection, ipilimumab, 1 mg
HCPCS	J9271	Injection, pembrolizumab, 1 mg
HCPCS	J9299	Injection, nivolumab, 1 mg
**General chemotherapy administration procedure codes**		
CPT	96401	Chemotherapy administration, subcutaneous or intramuscular; non-hormonal anti-neoplastic
CPT	96405	Chemotherapy administration; intralesional, up to and including 7 lesions
CPT	96409	Chemotherapy administration; intravenous, push technique, single or initial substance/drug
CPT	96411	Chemotherapy administration; intravenous, push technique, each additional substance/drug (List separately in addition to code for primary procedure)
CPT	96413	Chemotherapy administration, intravenous infusion technique; up to 1 hour, single or initial substance/drug

CPT	96415	Chemotherapy administration, intravenous infusion technique; each additional hour (List separately in addition to code for primary procedure)
CPT	96416	Chemotherapy administration, intravenous infusion technique; initiation of prolonged chemotherapy infusion (more than 8 hours), requiring use of a portable or implantable pump
CPT	96417	Chemotherapy administration, intravenous infusion technique; each additional sequential infusion (different substance/drug), up to 1 hour (List separately in addition to code for primary procedure)
HCPCS	G0498	Chemotherapy administration, intravenous infusion technique; initiation of infusion in the office/clinic setting using office/clinic pump/supplies, with continuation of the infusion in the community setting (e.g., home, domiciliary, rest home or assisted living) using a portable pump provided by the office/clinic, includes follow up office/clinic visit at the conclusion of the infusion
HCPCS	Q0084	Chemotherapy administration by infusion technique only, per visit
HCPCS	J9999	Antineoplastic drugs, not otherwise classified

Table S2. Patient Characteristics by First ICI treatment

	Fist ICI treatment																
	Atezolizumab		Avelumab		Cemiplimab		Durvalumab		Ipilimumab		Nivolumab		Pembrolizumab		Combo (nip+niv)		p-value
**Total, n**	**201**		**29**		**21**		**56**		**470**		**1766**		**2505**		**468**		
**Age at ICI Initiation**																	<.0001
18-44	**6**	3.0%	**1**	3.4%	**3**	14.3%	**0**	0.0%	**76**	16.2%	**140**	7.9%	**215**	8.6%	**86**	18.4%	
45-54	**17**	8.5%	**0**	0.0%	**1**	4.8%	**1**	1.8%	**95**	20.2%	**236**	13.4%	**321**	12.8%	**80**	17.1%	
55-64	**62**	30.8%	**9**	31.0%	**5**	23.8%	**18**	32.1%	**113**	24.0%	**546**	30.9%	**746**	29.8%	**156**	33.3%	
65-74	**77**	38.3%	**11**	37.9%	**4**	19.0%	**25**	44.6%	**127**	27.0%	**548**	31.0%	**771**	30.8%	**116**	24.8%	
>=75	**39**	19.4%	**9**	31.0%	**8**	38.1%	**12**	21.4%	**59**	12.6%	**296**	16.8%	**452**	18.0%	**30**	6.4%	
**Sex**																	<.0001
F	98	48.8%	13	44.8%	3	14.3%	21	37.5%	182	38.7%	749	42.4%	1086	43.4%	189	40.4%	
M	103	51.2%	16	55.2%	18	85.7%	35	62.5%	288	61.3%	1017	57.6%	1419	56.6%	279	59.6%	
**Hispanic**																	0.0354
Yes	**6**	3.0%	**0**	0.0%	**0**	0.0%	**0**	0.0%	**8**	1.7%	**58**	3.3%	**84**	3.4%	**10**	2.1%	
No	**184**	91.5%	**29**	100.0%	**20**	95.2%	**53**	94.6%	**454**	96.6%	**1544**	87.4%	**2316**	92.5%	**435**	92.9%	
Unknown	**11**	5.5%	**0**	0.0%	**1**	4.8%	**3**	5.4%	**8**	1.7%	**164**	9.3%	**105**	4.2%	**14**	3.0%	
**Race**																	<.0001
American Indian or Alaska Native	**0**	0.0%	**0**	0.0%	**0**	0.0%	**0**	0.0%	**0**	0.0%	**2**	0.1%	**7**	0.3%	**2**	0.4%	
Asian	**3**	1.5%	**0**	0.0%	**0**	0.0%	**0**	0.0%	**2**	0.4%	**23**	1.3%	**40**	1.6%	**4**	0.9%	
Black or African American	**20**	10.0%	**1**	3.4%	**0**	0.0%	**1**	1.8%	**4**	0.9%	**170**	9.6%	**171**	6.8%	**15**	3.2%	
Native Hawaiian/Other Pacific Islander	**0**	0.0%	**0**	0.0%	**0**	0.0%	**0**	0.0%	**0**	0.0%	**1**	0.1%	**5**	0.2%	**0**	0.0%	
Unknown	**10**	5.0%	**0**	0.0%	**2**	9.5%	**1**	1.8%	**9**	1.9%	**84**	4.8%	**120**	4.8%	**17**	3.6%	
White	**168**	83.6%	**28**	96.6%	**19**	90.5%	**54**	96.4%	**455**	96.8%	**1486**	84.1%	**2162**	86.3%	**430**	91.9%	
**Charlson Comorbidity Index[1]**																	<.0001
0	**51**	25.4%	**9**	31.0%	**9**	42.9%	**12**	21.4%	**263**	56.0%	**646**	36.6%	**903**	36.0%	210	44.9%	
1	**46**	22.9%	**9**	31.0%	**1**	4.8%	**16**	28.6%	**132**	28.1%	**442**	25.0%	**656**	26.2%	113	24.1%	

2	**50**	24.9%	**5**	17.2%	**1**	4.8%	**14**	25.0%	**39**	8.3%	**276**	15.6%	**429**	17.1%	67	14.3%	
>=3	**54**	26.9%	**6**	20.7%	**10**	47.6%	**14**	25.0%	**36**	7.7%	**402**	22.8%	**517**	20.6%	78	16.7%	
**Comorbidities[1]**																	
AIDS/HIV	**0**	0.0%	**0**	0.0%	**1**	4.8%	**0**	0.0%	**3**	0.6%	**18**	1.0%	**9**	0.4%	**2**	0.4%	0.0275
Cerebrovascular disease	**21**	10.4%	**5**	17.2%	**3**	14.3%	**8**	14.3%	**37**	7.9%	**170**	9.6%	**296**	11.8%	**45**	9.6%	0.0911
Congestive heart failure	**10**	5.0%	**0**	0.0%	**0**	0.0%	**1**	1.8%	**3**	0.6%	**62**	3.5%	**21**	0.8%	**68**	14.5%	0.0069
Chronic pulmonary disease	**70**	34.8%	**6**	20.7%	**4**	19.0%	**36**	64.3%	**59**	12.6%	**543**	30.7%	**685**	27.3%	**85**	18.2%	<.0001
Dementia	**0**	0.0%	**0**	0.0%	**1**	4.8%	**0**	0.0%	**0**	0.0%	**10**	0.6%	**20**	0.8%	**0**	0.0%	0.0246
Diabetes without chronic complication	**25**	12.4%	**5**	17.2%	**3**	14.3%	**8**	14.3%	**45**	9.6%	**148**	8.4%	**261**	10.4%	**42**	9.0%	0.1528
Diabetes with chronic complication	**19**	9.5%	**2**	6.9%	**4**	19.0%	**3**	5.4%	**11**	2.3%	**6**	0.3%	**137**	5.5%	**24**	5.1%	0.0015
Diabetes secondary to drug use	**7**	3.5%	**0**	0.0%	**1**	4.8%	**0**	0.0%	**1**	0.2%	**21**	1.2%	**28**	1.1%	**4**	0.9%	0.0149
Any diabetes[2]	**47**	23.4%	**7**	24.1%	**7**	33.3%	**11**	19.6%	**56**	11.9%	**255**	14.4%	**402**	16.0%	**67**	14.3%	0.001
Hemiplegia/paraplegia	**5**	2.5%	**0**	0.0%	**0**	0.0%	**2**	3.6%	**2**	0.4%	**34**	1.9%	**62**	2.5%	**9**	1.9%	0.1671
Metastatic solid tumor	**183**	91.0%	**25**	86.2%	**16**	76.2%	**43**	76.8%	**432**	91.9%	**1444**	81.8%	**2168**	86.5%	**427**	91.2%	<.0001
Myocardial infarction	**6**	3.0%	**1**	3.4%	**0**	0.0%	**1**	1.8%	**3**	0.6%	**57**	3.2%	**69**	2.8%	**9**	1.9%	0.1135
Mild liver disease	**56**	27.9%	**6**	20.7%	**2**	9.5%	**7**	12.5%	**83**	17.7%	**378**	21.4%	**528**	21.1%	**110**	23.5%	0.0405
Peptic ulcer disease	**4**	2.0%	**2**	6.9%	**0**	0.0%	**2**	3.6%	**5**	1.1%	**52**	2.9%	**72**	2.9%	**6**	1.3%	0.0889
Peripheral vascular disease	**31**	15.4%	**2**	6.9%	**2**	9.5%	**10**	17.9%	**28**	6.0%	**250**	14.2%	**339**	13.5%	**38**	8.1%	<.0001
Renal disease	**36**	17.9%	**4**	13.8%	**7**	33.3%	**4**	7.1%	**13**	2.8%	**228**	12.9%	**270**	10.8%	**55**	11.8%	<.0001
Rheumatic disease	**7**	3.5%	**0**	0.0%	**0**	0.0%	**3**	5.4%	**5**	1.1%	**40**	2.3%	**75**	3.0%	**12**	2.6%	0.166
Moderate/severe liver disease	**2**	1.0%	**1**	3.4%	**1**	4.8%	**0**	0.0%	**4**	0.9%	**63**	3.6%	**72**	2.9%	**6**	1.3%	0.0075
Hypertension	**108**	53.7%	**16**	55.2%	**10**	47.6%	**33**	58.9%	**144**	30.6%	**870**	49.3%	**1161**	46.3%	**184**	39.3%	<.0001
**Primary Cancer Site[3]**																	
Esophgeal	**3**	1.5%	**0**	0.0%	**0**	0.0%	**4**	7.1%	**0**	0.0%	**18**	1.0%	**74**	3.0%	**1**	0.2%	<.0001
Gastric	**3**	1.5%	**1**	3.4%	**0**	0.0%	**2**	3.6%	**6**	1.3%	**20**	1.1%	**95**	3.8%	**3**	0.6%	<.0001

Colon	**4**	2.0%	**0**	0.0%	**0**	0.0%	**2**	3.6%	**4**	0.9%	**47**	2.7%	**155**	6.2%	**11**	2.4%	<.0001
Liver	**11**	5.5%	**1**	3.4%	**0**	0.0%	**0**	0.0%	**6**	1.3%	**152**	8.6%	**150**	6.0%	**16**	3.4%	<.0001
Hodgekin's	**0**	0.0%	**1**	3.4%	**0**	0.0%	**0**	0.0%	**0**	0.0%	**36**	2.0%	**13**	0.5%	**1**	0.2%	<.0001
Melanoma	**5**	2.5%	**8**	27.6%	**6**	28.6%	**1**	1.8%	**462**	98.3%	**445**	25.2%	**650**	25.9%	**282**	60.3%	<.0001
Lung	**101**	50.2%	**10**	34.5%	**0**	0.0%	**53**	94.6%	**21**	4.5%	**736**	41.7%	**963**	38.4%	**84**	17.9%	<.0001
Renal Cell Carcinoma	**21**	10.4%	**1**	3.4%	**0**	0.0%	**0**	0.0%	**2**	0.4%	**248**	14.0%	**89**	3.6%	**106**	22.6%	<.0001
Bladder	**65**	32.3%	**0**	0.0%	**0**	0.0%	**2**	3.6%	**1**	0.2%	**74**	4.2%	**189**	7.5%	**8**	1.7%	<.0001
Markel Cell Carcinoma	**0**	0.0%	**11**	37.9%	**0**	0.0%	**0**	0.0%	**1**	0.2%	**7**	0.4%	**15**	0.6%	**1**	0.2%	<.0001
Head and Neck	**10**	5.0%	**6**	20.7%	**5**	23.8%	**1**	1.8%	**9**	1.9%	**146**	8.3%	**219**	8.7%	**14**	3.0%	<.0001
Breast	**31**	15.4%	**2**	6.9%	**0**	0.0%	**0**	0.0%	**11**	2.3%	**59**	3.3%	**141**	5.6%	**10**	2.1%	<.0001
Cervix	**0**	0.0%	**0**	0.0%	**0**	0.0%	**0**	0.0%	**2**	0.4%	**10**	0.6%	**39**	1.6%	**3**	0.6%	0.0187
Primary mediastinal (thymic) large B-cell lymphoma	**0**	0.0%	**0**	0.0%	**0**	0.0%	**0**	0.0%	**1**	0.2%	**1**	0.1%	**0**	0.0%	**0**	0.0%	0.6074
Other	**9**	4.5%	**2**	6.9%	**12**	57.1%	**0**	0.0%	**6**	1.3%	**117**	6.6%	**291**	11.6%	**14**	3.0%	<.0001
**Prior Cancer Treatment**																	
**Radiation(Y/N)**	**63**	31.3%	**15**	51.7%	**6**	28.6%	**45**	80.4%	**108**	23.0%	**626**	35.4%	**887**	35.4%	**123**	26.3%	<.0001
**Chemotherapy (Y/N)**	**59**	29.4%	**4**	13.8%	**0**	0.0%	**26**	46.4%	**26**	5.5%	**575**	32.6%	**496**	19.8%	**38**	8.1%	<.0001
Alkylating Agent	**45**	22.4%	**4**	13.8%	**0**	0.0%	**26**	46.4%	**16**	3.4%	**495**	28.0%	**407**	16.2%	**34**	7.3%	<.0001
Antimetabolite	**23**	11.4%	**1**	3.4%	**0**	0.0%	**3**	5.4%	**3**	0.6%	**305**	17.3%	**235**	9.4%	**9**	1.9%	<.0001
Antimitotic Agent	**19**	9.5%	**2**	6.9%	**0**	0.0%	**16**	28.6%	**5**	1.1%	**213**	12.1%	**4**	0.2%	**194**	41.5%	<.0001
Antitumor Antibiotic	**18**	9.0%	**0**	0.0%	**0**	0.0%	**1**	1.8%	**3**	0.6%	**84**	4.8%	**92**	3.7%	**3**	0.6%	<.0001
Enzyme Inhibitor	**0**	0.0%	**0**	0.0%	**0**	0.0%	**0**	0.0%	**8**	1.7%	**0**	0.0%	**0**	0.0%	**0**	0.0%	0.0173
Plant Alkaloid	**15**	7.5%	**2**	6.9%	**0**	0.0%	**10**	17.9%	**0**	0.0%	**75**	4.2%	**36**	1.4%	**28**	6.0%	<.0001
Proteasome Inhibitor	**0**	0.0%	**0**	0.0%	**0**	0.0%	**0**	0.0%	**4**	0.9%	**0**	0.0%	**6**	0.2%	**0**	0.0%	0.8914
Topoisomerase I Inhibitor	**4**	2.0%	**0**	0.0%	**0**	0.0%	**0**	0.0%	**1**	0.2%	**25**	1.4%	**34**	1.4%	**8**	1.7%	0.3982
Chemotherapy - non specific	**1**	0.5%	**0**	0.0%	**0**	0.0%	**0**	0.0%	**6**	1.3%	**6**	0.3%	**24**	1.0%	**1**	0.2%	0.159

Other irAEs developed within 1 year of ICI initiation[4]

Hematologic	**109**	54.2%	**13**	44.8%	**5**	23.8%	**19**	33.9%	**177**	37.7%	**758**	42.9%	**1140**	45.5%	**172**	36.8%	<.0001
Pulmonary (pneumonitis)	**55**	27.4%	**9**	31.0%	**1**	4.8%	**28**	50.0%	**79**	16.8%	**436**	24.7%	**629**	25.1%	**110**	23.5%	<.0001
Endocrine	**88**	43.8%	**11**	37.9%	**7**	33.3%	**19**	33.9%	**228**	48.5%	**770**	43.6%	**1103**	44.0%	**252**	53.8%	0.0011
Renal (acute kidney injury)	**56**	27.9%	**6**	20.7%	**0**	0.0%	**9**	16.1%	**107**	22.8%	**360**	20.4%	**553**	22.1%	**127**	27.1%	0.0036
Neurological	**68**	33.8%	**10**	34.5%	**4**	19.0%	**13**	23.2%	**87**	18.5%	**409**	23.2%	**739**	29.5%	**103**	22.0%	<.0001
Hepatic	**44**	21.9%	**6**	20.7%	**2**	9.5%	**11**	19.6%	**95**	20.2%	**286**	16.2%	**510**	20.4%	**137**	29.3%	<.0001
Gastrointestinal	**44**	21.9%	**8**	27.6%	**2**	9.5%	**5**	8.9%	**144**	30.6%	**384**	21.7%	**553**	22.1%	**131**	28.0%	<.0001
Skin(vitiligo)	**3**	1.5%	**0**	0.0%	**0**	0.0%	**1**	1.8%	**78**	16.6%	**102**	5.8%	**180**	7.2%	**55**	11.8%	<.0001

1. Comorbidities and Charlson Cormobidity Index (CCI) were calculated based on diagnoses before or on the day of ICI initiation. ICD 9 and 10 codes used to calculate the CCI were from Quan et al. (2005). Primary cancer diagnoses were not included in the calculation of the index but metastatic/secondary cancers were included. Hierarchy coding was applied to prevent duplicated accounting. For instance, if a person had diagnoses of both a mild and a severe form of the disease (e.g. mild and moderate/severe liver disease, diabetes with and without chronic complications), the patient was only scored on the more severe disease in the CCI.
2. Any diabetes included patients who had any of the following diagnoses, diabetes without chronic complicaiton, diabetes with chronic complication, or diabetes secondary to drug use, prior to or on the day of ICI initiation.
3. Primary can sites are based on diagnoses of primary cancers (excluding secondary diagnoses) before ICI initiation or 30 days after. Site variables are not mutually exclusive and patients may have multiple cancer sites.
4. Other irAEs included hematologic [anemia, thrombocytopenia, leukopenia], pneumonitis, endocrine [hypothyroidism, hyperthyroidism, hypophysitis/PGA, Hyper/hypo-parathyroidism, diabetes type I, dysfunctional uterine bleeding/infertility], renal [acute kidney injury/AKI], neurological [encephalitis/myelitis/encephalomyelitis, neuritis, meningitis], hepatic [hepatitis], gastrointestinal (GI) [colitis, pancreatitis, mucositis] and skin [vitiligo]).

Table S3 Patient Characteristics by Primary Cancer Site

	Primary Cancer Site[1]													
	Lung		Melanoma		RCC		Urothelial		Head & Neck		MCC		Liver	
**Total, *n***	**1968**		**1861**		**467**		**339**		**410**		**35**		**336**	
**Age at ICI Initiation**														
18-44	**74**	3.8%	**286**	15.4%	**20**	4.3%	**6**	1.8%	**24**	5.9%	**0**	0.0%	**23**	6.8%
45-54	**188**	9.6%	**301**	16.2%	**64**	13.7%	**32**	9.4%	**61**	14.9%	**1**	2.9%	**42**	12.5%
55-64	**662**	33.6%	**470**	25.3%	**139**	29.8%	**83**	24.5%	**138**	33.7%	**2**	5.7%	**118**	35.1%
65-74	**686**	34.9%	**513**	27.6%	**158**	33.8%	**115**	33.9%	**117**	28.5%	**16**	45.7%	**107**	31.8%
>=75	**358**	18.2%	**291**	15.6%	**86**	18.4%	**103**	30.4%	**70**	17.1%	**16**	45.7%	**46**	13.7%
**Sex**														
F	**957**	48.6%	**707**	38.0%	**151**	32.3%	**99**	29.2%	**97**	23.7%	**10**	28.6%	**123**	36.6%
M	**1011**	51.4%	**1154**	62.0%	**316**	67.7%	**240**	70.8%	**313**	76.3%	**25**	71.4%	**213**	63.4%
**Hispanic**														
Yes	**48**	2.4%	**39**	2.1%	**22**	4.7%	**13**	3.8%	**15**	3.7%	**2**	5.7%	**17**	5.1%
No	**1782**	90.5%	**1761**	94.6%	**425**	91.0%	**310**	91.4%	**365**	89.0%	**31**	88.6%	**295**	87.8%
Unknown	**138**	7.0%	**61**	3.3%	**20**	4.3%	**16**	4.7%	**30**	7.3%	**2**	5.7%	**24**	7.1%
**Race**														
American Indian or Alaska Native	**3**	0.2%	**2**	0.1%	**2**	0.4%	**1**	0.3%	**2**	0.5%	**0**	0.0%	**11**	3.3%
Asian	**29**	1.5%	**7**	0.4%	**5**	1.1%	**21**	6.2%	**8**	2.0%	**0**	0.0%	**44**	13.1%
Black or African American	**233**	11.8%	**10**	0.5%	**36**	7.7%	**0**	0.0%	**27**	6.6%	**0**	0.0%	**0**	0.0%
Native Hawaiian/Other Pacific Islander	**3**	0.2%	**0**	0.0%	**0**	0.0%	**0**	0.0%	**0**	0.0%	**0**	0.0%	**0**	0.0%
Unknown	**67**	3.4%	**51**	2.7%	**26**	5.6%	**21**	6.2%	**22**	5.4%	**4**	11.4%	**19**	5.7%
White	**1633**	83.0%	**1791**	96.2%	**398**	85.2%	**296**	87.3%	**351**	85.6%	**31**	88.6%	**262**	78.0%
**Charlson Comorbidity Index[2]**														
0	**564**	28.7%	**979**	52.6%	**120**	25.7%	**85**	25.1%	**142**	34.6%	**4**	11.4%	**22**	6.5%
1	**568**	28.9%	**465**	25.0%	**84**	18.0%	**60**	17.7%	**116**	28.3%	**13**	37.1%	**101**	30.1%

2	**384**	19.5%	**222**	11.9%	**85**	18.2%	**70**	20.6%	**63**	15.4%	**12**	34.3%	**51**	15.2%
>=3	**452**	23.0%	**195**	10.5%	**178**	38.1%	**124**	36.6%	**89**	21.7%	**6**	17.1%	**162**	48.2%
**Comorbidities[2]**														
AIDS/HIV	**13**	0.7%	**10**	0.5%	**3**	0.6%	**1**	0.3%	**3**	0.7%	**1**	2.9%	**4**	1.2%
Cerebrovascular disease	**266**	13.5%	**175**	9.4%	**36**	7.7%	**33**	9.7%	**59**	14.4%	**8**	22.9%	**37**	11.0%
Congestive heart failure	**78**	4.0%	**29**	1.6%	**25**	5.4%	**18**	5.3%	**13**	3.2%	**35**	100.0%	**14**	4.2%
Chronic pulmonary disease	**910**	46.2%	**256**	13.8%	**94**	20.1%	**84**	24.8%	**110**	26.8%	**6**	17.1%	**89**	26.5%
Dementia	**12**	0.6%	**6**	0.3%	**3**	0.6%	**3**	0.9%	**4**	1.0%	**36**	102.9%	**2**	0.6%
Diabetes without chronic complication	**207**	10.5%	**157**	8.4%	**47**	10.1%	**35**	10.3%	**51**	12.4%	**7**	20.0%	**45**	13.4%
Diabetes with chronic complication	**113**	5.7%	**55**	3.0%	**58**	12.4%	**39**	11.5%	**19**	4.6%	**2**	5.7%	**31**	9.2%
Diabetes secondary to drug use	**27**	2.5%	**12**	0.6%	**6**	1.3%	**6**	1.8%	**3**	0.7%	**1**	2.9%	**6**	1.8%
Any diabetes[3]	**324**	16.5%	**215**	11.6%	**105**	22.5%	**74**	21.8%	**70**	17.1%	**10**	28.6%	**76**	22.6%
Hemiplegia/paraplegia	**49**	2.5%	**20**	1.1%	**15**	3.2%	**9**	2.7%	**14**	3.4%	**0**	0.0%	**3**	0.9%
Metastatic solid tumor	**1682**	85.5%	**1697**	91.2%	**428**	91.6%	**307**	90.6%	**377**	92.0%	**26**	74.3%	**267**	12.0%
Myocardial infarction	**67**	3.4%	**35**	1.9%	**16**	3.4%	**18**	5.3%	**11**	2.7%	**1**	2.9%	**12**	3.6%
Mild liver disease	**397**	20.2%	**358**	19.2%	**136**	29.1%	**73**	21.5%	**78**	19.0%	**5**	14.3%	**183**	54.5%
Peptic ulcer disease	**53**	2.7%	**21**	1.1%	**14**	3.0%	**8**	2.4%	**11**	2.7%	**1**	2.9%	**19**	5.7%
Peripheral vascular disease	**337**	17.1%	**156**	8.4%	**71**	15.2%	**59**	17.4%	**75**	18.3%	**3**	8.6%	**52**	15.5%
Renal disease	**187**	9.5%	**94**	5.1%	**191**	40.9%	**131**	38.6%	**42**	10.2%	**9**	25.7%	**35**	10.4%
Rheumatic disease	**59**	3.0%	**47**	2.5%	**12**	2.6%	**9**	2.7%	**9**	2.2%	**4**	11.4%	**11**	3.3%
Moderate/severe liver disease	**20**	1.0%	**13**	0.7%	**5**	1.1%	**9**	2.7%	**5**	1.2%	**0**	0.0%	**102**	30.4%
Hypertension	**985**	50.1%	**669**	35.9%	**318**	68.1%	**208**	61.4%	**200**	48.8%	**22**	62.9%	**193**	57.4%
**Prior Cancer Treatment**														
**Radiation(Y/N)**	**906**	46.0%	**346**	18.6%	**139**	29.8%	**97**	28.6%	**248**	60.5%	**16**	45.7%	**104**	31.0%
**Chemotherapy (Y/N)**	**601**	30.5%	**57**	3.1%	**55**	11.8%	**114**	33.6%	**152**	37.1%	**3**	8.6%	**112**	33.3%

Alkylating Agent	**561**	28.5%	**37**	2.0%	**34**	7.3%	**86**	25.4%	**138**	33.7%	**2**	5.7%	**60**	17.9%
Antimetabolite	**289**	14.7%	**16**	0.9%	**31**	6.6%	**78**	23.0%	**46**	11.2%	**1**	2.9%	**43**	12.8%
Antimitotic Agent	**260**	13.2%	**15**	0.8%	**15**	3.2%	**19**	5.6%	**55**	13.4%	**1**	2.9%	**25**	7.4%
Antitumor Antibiotic	**20**	1.0%	**8**	0.4%	**20**	4.3%	**43**	12.7%	**12**	2.9%	**0**	0.0%	**54**	16.1%
Enzyme Inhibitor	**3**	0.2%	**0**	0.0%	**5**	1.1%	**1**	0.3%	**0**	0.0%	**0**	0.0%	**1**	0.3%
Plant Alkaloid	**114**	5.8%	**3**	0.2%	**7**	1.5%	**9**	2.7%	**8**	2.0%	**1**	2.9%	**9**	2.7%
Proteasome Inhibitor	**0**	0.0%	**0**	0.0%	**1**	0.2%	**0**	0.0%	**0**	0.0%	**0**	0.0%	**1**	0.3%
Topoisomerase I Inhibitor	**15**	0.8%	**1**	0.1%	**2**	0.4%	**1**	0.3%	**1**	0.2%	**0**	0.0%	**14**	4.2%
Chemotherapy - non specific	**6**	0.3%	**12**	0.6%	**1**	0.2%	**2**	0.6%	**0**	0.0%	**0**	0.0%	**1**	0.3%
**First ICI treatment**														
Atezolizumab	**101**	5.1%	**5**	0.3%	**21**	4.5%	**65**	19.2%	**10**	2.4%	**0**	0.0%	**11**	3.3%
Avelumab	**10**	0.5%	**8**	0.4%	**1**	0.2%	**0**	0.0%	**6**	1.5%	**11**	31.4%	**1**	0.3%
Cemiplimab	**0**	0.0%	**6**	0.3%	**0**	0.0%	**0**	0.0%	**5**	1.2%	**0**	0.0%	**0**	0.0%
Durvalumab	**53**	2.7%	**1**	0.1%	**0**	0.0%	**2**	0.6%	**1**	0.2%	**0**	0.0%	**0**	0.0%
Ipilimumab	**21**	1.1%	**462**	24.8%	**2**	0.4%	**1**	0.3%	**9**	2.2%	**1**	2.9%	**6**	1.8%
Nivolumab	**736**	37.4%	**445**	23.9%	**248**	53.1%	**74**	21.8%	**146**	35.6%	**7**	20.0%	**152**	45.2%
Combo (niv+ipi)	**84**	4.3%	**282**	15.2%	**106**	22.7%	**8**	2.4%	**14**	3.4%	**1**	2.9%	**16**	4.8%
Pembrolizumab	**963**	48.9%	**650**	34.9%	**89**	19.1%	**189**	55.8%	**219**	53.4%	**15**	42.9%	**150**	44.6%
**Other irAEs developed within 1 year of ICI initiation[4]**										0.0%				0.0%
Hematologic	**886**	45.0%	**569**	30.6%	**220**	47.1%	**198**	58.4%	**180**	43.9%	**18**	51.4%	**191**	56.8%
Pulmonary (pneumonitis)	**741**	37.7%	**294**	15.8%	**110**	23.6%	**62**	18.3%	**118**	28.8%	**5**	14.3%	**62**	18.5%
Endocrine	**899**	45.7%	**835**	44.9%	**263**	56.3%	**133**	39.2%	**204**	49.8%	**19**	54.3%	**130**	38.7%
Renal (acute kidney injury)	**381**	19.4%	**316**	17.0%	**198**	42.4%	**162**	47.8%	**80**	19.5%	**5**	14.3%	**90**	26.8%
Neurological	**552**	28.0%	**339**	18.2%	**107**	22.9%	**109**	32.2%	**125**	30.5%	**10**	28.6%	**77**	22.9%
Hepatic	**295**	15.0%	**424**	22.8%	**105**	22.5%	**66**	19.5%	**60**	14.6%	**7**	20.0%	**121**	36.0%
Gastrointestinal	**395**	20.1%	**421**	22.6%	**134**	28.7%	**72**	21.2%	**150**	36.6%	**9**	25.7%	**78**	23.2%

Skin(vitiligo)	**65**	3.3%	**295**	15.9%	**21**	4.5%		**8**	2.4%	**25**	6.1%		**2**	5.7%	**13**	3.9%

1. Primary can sites are based on diagnoses of primary cancers (excluding secondary diagnoses) before ICI initiation or 30 days after. Site variables are not mutually exclusive and patients may have multiple cancer sites and could not be compared.
2. Comorbidities and Charlson Cormobidity Index (CCI) were calculated based on diagnoses before or on the day of ICI initiation. ICD 9 and 10 codes used to calculate the CCI were from Quan et al. (2005). Primary cancer diagnoses were not included in the calculation of the index but metastatic/secondary cancers were included. Hierarchy coding was applied to prevent duplicated accounting. For instance, if a person had diagnoses of both a mild and a severe form of the disease (e.g. mild and moderate/severe liver disease, diabetes with and without chronic complications), the patient was only scored on the more severe disease in the CCI.
3. Any diabetes included patients who had any of the following diagnoses, diabetes without chronic complicaiton, diabetes with chronic complication, or diabetes secondary to drug use, prior to or on the day of ICI initiation.
4. Other irAEs included hematologic [anemia, thrombocytopenia, leukopenia], pneumonitis, endocrine [hypothyroidism, hyperthyroidism, hypophysitis/PGA, Hyper/hypo-parathyroidism, diabetes type I, dysfunctional uterine bleeding/infertility], renal [acute kidney injury/AKI], neurological [encephalitis/myelitis/encephalomyelitis, neuritis, meningitis], hepatic [hepatitis], gastrointestinal (GI) [colitis, pancreatitis, mucositis] and skin [vitiligo]).

Table S4. Estimated Rate per Person-Year

		CAEs within One Year		Follow-up time (in years)					
	n	Yes	%	Median	IQR	Total Person-Year	Rate per Person-Year	95% CI	
Any CAE	5518	691	12.5%	0.647	(0.252-1)	3394.4	0.203	0.19	0.22
Arrhythmia	5518	511	9.3%	0.647	(0.252-1)	3394.4	0.150	0.14	0.16
Acute MI	5518	94	1.7%	0.647	(0.252-1)	3394.4	0.028	0.02	0.03
Myocarditis	5518	116	2.1%	0.647	(0.252-1)	3394.4	0.034	0.03	0.04
Pericarditis	5518	68	1.2%	0.647	(0.252-1)	3394.4	0.020	0.02	0.03
Cardiomyopathy	5518	47	0.9%	0.647	(0.252-1)	3394.4	0.014	0.01	0.02
		**Overall Death**		**Follow-up time (in years)**					
	**N**	**Yes**	**%**	**Median**	**IQR**	**Total Person-Year**	**Rate Per Person-Year**	**95% CI**	
All	5518	2070	37.5%	0.647	(0.252-1.441)	5786.2	0.358	0.343	0.373
CAE within 1 Year (Yes)	691	380	55.0%	0.682	(0.301-1.230)	679.6	0.331	0.316	0.347
CAE within 1 Year (No)	4827	1690	35.0%	0.636	(0.249-1.477)	5106.7	0.559	0.506	0.618
